# The binding mode of orphan glycyl-tRNA synthetase with tRNA supports the synthetase classification and reveals large domain movements

**DOI:** 10.1126/sciadv.adf1027

**Published:** 2023-02-08

**Authors:** Lu Han, Zhiteng Luo, Yingchen Ju, Bingyi Chen, Taotao Zou, Junjian Wang, Jun Xu, Qiong Gu, Xiang-Lei Yang, Paul Schimmel, Huihao Zhou

**Affiliations:** ^1^ Guangdong Provincial Key Laboratory of Chiral Molecule and Drug Discovery, School of Pharmaceutical Sciences, Sun Yat-sen University, Guangzhou 510006, China.; ^2^ Research Center for Drug Discovery, School of Pharmaceutical Sciences, Sun Yat-sen University, Guangzhou 510006, China.; ^3^ Department of Molecular Medicine, The Scripps Research Institute, La Jolla, CA 92037, USA.

## Abstract

As a class of essential enzymes in protein translation, aminoacyl–transfer RNA (tRNA) synthetases (aaRSs) are organized into two classes of 10 enzymes each, based on two conserved active site architectures. The (αβ)_2_ glycyl-tRNA synthetase (GlyRS) in many bacteria is an orphan aaRS whose sequence and unprecedented X-shaped structure are distinct from those of all other aaRSs, including many other bacterial and all eukaryotic GlyRSs. Here, we report a cocrystal structure to elucidate how the orphan GlyRS kingdom specifically recognizes its substrate tRNA. This structure is sharply different from those of other aaRS-tRNA complexes but conforms to the clash-free, cross-class aaRS-tRNA docking found with conventional structures and reinforces the class-reconstruction paradigm. In addition, noteworthy, the X shape of orphan GlyRS is condensed with the largest known spatial rearrangement needed by aaRSs to capture tRNAs, which suggests potential nonactive site targets for aaRS-directed antibiotics, instead of less differentiated hard-to-drug active site locations.

## Teaser

Cocrystal structure of orphan GlyRS-tRNA complex provides insights into aaRS classifications and antibiotics discovery.

## INTRODUCTION

The functions of aminoacyl–transfer RNA (tRNA) synthetases (aaRSs) in protein biosynthesis are so essential that the members in this enzyme family are believed to have appeared before the last universal common ancestor and evolved thereafter (*1*, *2*). On the basis of their active site architectures, aaRSs are divided into two classes of 10 enzymes each (*3*), which are further divided into three subclasses (a, b, and c) depending on the closeness of the evolutionary relationships (*4*). The early work of Rodin and Ohno (*5*) and further works by Carter *et al*. (*6*–*8*) on urzymes revealed that two classes of aaRSs might arise from opposite strands of early RNA genomes, and they were found to approach the acceptor stem of tRNA from opposite sides (*9*). Specially, when members of class IIa are bound to tRNA, they do so in a way that allows members of class Ia to also fit onto the same tRNA with no steric clashes. However, class IIa members cannot be paired with members of class Ib or Ic. Thus, Ia enzymes specifically pair with IIa, Ib enzymes with IIb, and Ic enzymes with IIc (*4*, *10*). Two types (class Ib or IIb) of lysyl-tRNA synthetase (LysRS) have been found in different organisms, and they can be simultaneously docked to tRNA^Lys^ without steric crashes (*11*, *12*). These observations, and other considerations based on the sequences of active site regions, gave rise to the hypothesis that early aaRSs bound in pairs to protect each tRNA from degradation by heat or nucleases (*10*). This hypothesis is generally consistent with all the aaRSs whose tRNA binding modes have been clarified.

In most bacteria, the class II glycyl-tRNA synthetase (GlyRS) in charge of the synthesis of glycyl-tRNA^Gly^ is a special member of aaRS family (*13*). While GlyRS in eukaryotes, archaea, and some bacteria is a homodimer like most class II aaRSs (*14*, *15*), GlyRS in most bacteria is a unique (αβ)_2_ heterotetramer (*13*, *16*). Unlike other aaRSs, where the ancestral relationship between them can be clearly seen through evolution, in sequence and structure, the (αβ)_2_ GlyRS is distinct from any other aaRS, including other GlyRSs. Thus, it is a unique orphan aaRS.

Recently, crystal structures of *Escherichia coli* GlyRS and *Thermanaerothrix daxensis* GlyRS were solved as representative (αβ)_2_ orphan GlyRSs (*12*, *17*). Orphan GlyRS adopts an unprecedented X-shaped architecture with two α subunits forming a globe at the center and two β subunits flanking the two sides of the α subunit dimer. Regarding its unusual structure, when we docked in silico the orphan GlyRS to its tRNA partner, we found that, for this docking model, there was no steric clash with the way that subclass Ia enzyme fits onto the tRNA (*12*). This result provided further support to the hypothesis for the origin of the aaRS classes (*4*, *5*, *10*, *18*, *19*). In addition, this foundation provided the basis for the present work, which was motivated by the obvious need to obtain a cocrystal of the unique GlyRS with bound tRNA/s to further test the hypothesis about the origin of the two classes of aaRSs.

Furthermore, we wanted to find at least a starting path toward using the unique X-shaped GlyRS to exploit for badly needed new antibiotics. Because of being essential proteins encoded by single genes, aaRSs have long been targets for development of antibiotics (*20*). Both active and editing sites have been probed for this purpose. Success was achieved with a potent leucyl-tRNA synthetase (LeuRS) editing site inhibitor that was eventually developed into a U.S. Food and Drug Administration–approved antifungal agent (tavaborole) (*21*). Much earlier, the isoleucyl-tRNA synthetase (IleRS) active site–directed natural product mupirocin was developed as an approved and widely used antibacterial (*22*). Further developments have been hindered by several factors, not the least of which is achieving species and aaRS specificity. The specificity problems are difficult because the active sites of aaRSs across evolution are built on either the well conserved class I or class II architectural framework. However, the unusual structure of the (αβ)_2_ orphan GlyRS in pathogenic bacteria suggested an opportunity to exploit its well-differentiated (from host GlyRS and all other aaRSs) unique structure for antibiotic development. Notably, the recognition of substrate tRNA^Gly^ by GlyRS has species specificity. Bacterial (αβ)_2_ GlyRS can only aminoacylate tRNA^Gly^ molecules from bacteria but not those from eukaryotes (*23*). The best-known discriminator base is nucleotide 73, which is conserved as U in prokaryotic tRNA^Gly^ and as A in eukaryotic tRNA^Gly^, respectively (*23*, *24*). The kingdom-specific GlyRS-tRNA interactions provide a valuable chance for developing bacteria-selective inhibitors, but the detailed information about how (αβ)_2_ GlyRS recognizes and charges bacterial tRNA^Gly^ is needed.

Here, we report a cocrystal structure of *E. coli* orphan GlyRS bound with two tRNA^Gly^ and two intermediate adenylate-like analogs, 2-chloro-5′-O-[*N*-(glycyl)sulfamoyl] adenosine (GlySA). The structure mimics the aminoacylation state in that the 3′ CCA end of tRNA^Gly^ extends with a unique conformation into the active site cavity to approach GlySA, awaiting to receive the activated glycine. Compared with the tRNA-free state, a large rotation of the HD domain (related to a hydrolase superfamily with a conserved histidine-aspartate catalytic doublet) (*25*), together with a large movement of the anticodon binding domain (ABD) of the β subunit, was observed upon tRNA^Gly^ binding. A loop in the HD domain directly interacts with the critical discriminator base U73. By revealing how the enzyme fits onto its tRNAs, the cocrystal structure further supports the hypothesis of the origin of the aaRS classes. Moreover, drugging a potential nonactive site to prevent the CCA end of tRNA from accessing the active site may be an alternative way to work around the difficulties associated with the more conventional active site–directed inhibitors.

## RESULTS

### The orphan *Ec*GlyRS is cocrystallized with two bound tRNA^Gly^

**Fig. 1.**
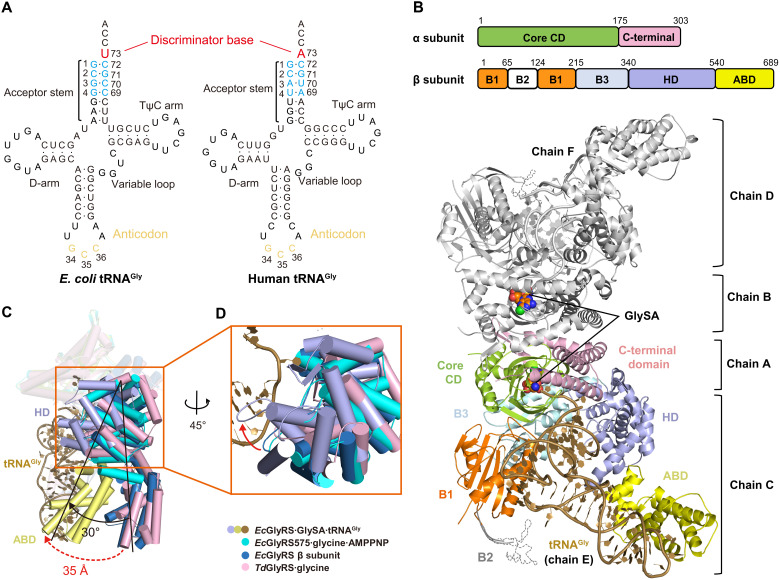
The overall view of orphan *Ec*GlyRS∙GlySA∙tRNA^Gly^ ternary complex. (**A**) Cloverleaf models of *E. coli* and human tRNA^Gly^(GCC). (**B**) Cartoon representation of the overall structure of orphan (αβ)_2_ *Ec*GlyRS in complex with *E. coli* tRNA^Gly^(GCC) and an intermediate product analog GlySA. The protomer consisting of chains A and C is colored the same as domain diagram above, and the bound tRNA^Gly^ (chain E) is colored in dark yellow. The other protomer (chains B and D) and its corresponding substrate tRNA^Gly^ (chain F) are colored in gray. GlySA is presented as spheres in both active site cavities. (**C**) Structural comparison of *Ec*GlyRS∙GlySA∙tRNA^Gly^ complex, *Ec*GlyRS575∙glycine∙AMPPNP complex (cyan) (PDB code 7eiv), *Ec*GlyRS β subunit predicted by AlphaFold2 (blue) (www.alphafold.ebi.ac.uk/entry/P00961), and *Td*GlyRS∙glycine (pink) (PDB code 7lu4) complex reveals that major conformation changes occur at the C-terminal part of β subunit. HD and ABD rotate about 30° upon tRNA binding, and it brings ABD move inward about 35 Å to make contacts with the anticodon loop of tRNA^Gly^. (**D**) A large rotation of HD domain for clamping the major groove of tRNA^Gly^ acceptor stem.

To understand how (αβ)_2_ heterotetrameric orphan GlyRS recognizes and charges its kingdom-specific substrate tRNA^Gly^, we determined a cocrystal structure of *E. coli* GlyRS (*Ec*GlyRS) in complex with *Ec*tRNA^Gly^(GCC) (Fig. 1A) and an intermediate product analog GlySA (*26*) at 2.9 Å with *R*/*R*_free_ = 0.230/0.260 (table S1). The asymmetric unit contains one *Ec*GlyRS∙GlySA∙tRNA^Gly^ ternary complex consisting of an (αβ)_2_ *Ec*GlyRS, two GlySA, and two tRNA^Gly^ molecules with a total molecular weight of about 280 kDa (Fig. 1B). *Ec*GlyRS is organized as two protomers (one α subunit plus one β subunit), which are symmetric to each other through a noncrystallographic twofold rotation axis for both their structure and tRNA binding modes (Fig. 1B and fig. S1). Because of the better density map (fig. S2A), the protomer consisting of chains A (α subunit) and C (β subunit) and the corresponding substrate tRNA^Gly^ (chain E) were used in the following structural analysis unless otherwise indicated.

The α subunit consists of a core catalytic domain (CD) and a C-terminal domain, and the β subunit could be divided into five domains named as B1, B2, B3, HD, and ABD. While the anticodon loop of tRNA^Gly^ is recognized by ABD, the acceptor stem binds to the α subunit and HD domain of the β subunit (from the same protomer). The 3′ terminal CCA end of tRNA^Gly^ extends into the active site (Fig. 1B). Notably, on the basis of structural and mutagenesis analysis, the B2 domain was previously speculated to contribute to the recognition of tRNA^Gly^ through interacting with the elbow region of tRNA^Gly^ (*12*). However, the electron density of the B2 domain is weak (because of its dynamics) that this interaction could not be observed in our *Ec*GlyRS∙GlySA∙tRNA^Gly^ ternary complex structure. It is likely that the capture of tRNA^Gly^ by *Ec*GlyRS involves multiple steps, and the B2 domain may only interact with tRNA^Gly^ at a step other than the state captured in the crystal. Human GlyRS (*Hs*GlyRS) also has a similar domain named Ins3 that recognizes the elbow region of human tRNA^Gly^, and a large movement of Ins3 was observed between different steps of catalysis (*27*). The Ins3 domain facilitating tRNA binding is coming from the other subunit of homodimeric *Hs*GlyRS, and this cross-subunit/protomer tRNA binding manner is commonly used in class II aaRSs (*28*–*30*). In contrast, our structure indicates that the two protomers of (αβ)_2_ GlyRS recognize and capture their substrate tRNA^Gly^ independently.

### The C-terminal part of the β subunit undergoes a large rotation and movement upon tRNA^Gly^ binding

To clarify the structural movements of *Ec*GlyRS required for tRNA binding, the structure of the *Ec*GlyRS∙GlySA∙tRNA^Gly^ complex was superimposed on that of the *Ec*GlyRS575∙glycine∙AMPPNP complex [Protein Data Bank (PDB) code 7eiv] (*12*), *Td*GlyRS∙glycine complex (PDB code 7lu4) (*17*), and full-length *Ec*GlyRS β subunit (predicted by AlphaFold2; www.alphafold.ebi.ac.uk/entry/P00961) (*31*). Compared with those GlyRS structures without tRNA binding, a large domain rotation and movement in the *Ec*GlyRS∙GlySA∙tRNA^Gly^ complex occurs at the C-terminal part of the β subunit (HD and ABD) (Fig. 1C). Notably, the C-terminal parts of the β subunits in GlyRS structures without bound tRNA^Gly^ all have a similar orientation to each other (Fig. 1C). This observation supports that the large domain rotation of GlyRS in the existence of tRNA has a particular biological relevance. This rotation of about 30° clamps tRNA in the *Ec*GlyRS∙GlySA∙tRNA^Gly^ complex (Fig. 1, C and D). It happens mainly at the linker between the B3 and HD domains (fig. S3A), with the HD and ABD domains rotating as a whole (fig. S3B). As a result, the ABD moves inward about 35 Å to make contact with the anticodon loop of tRNA^Gly^ in *Ec*GlyRS∙GlySA∙tRNA^Gly^ complex (Fig. 1C).

Large domain movements upon tRNA binding occur in some other aaRSs. For class I aaRSs, the largest structural movement induced by tRNA binding is the C-terminal domain of tyrosyl-tRNA synthetase (TyrRS), which moves about 25 Å to contact and recognize the long variable loop of tRNA^Tyr^ (fig. S4A) (*32*). For class II aaRSs, the Ins3 domain of human GlyRS and the C-Ala domain of alanyl-tRNA synthetase (AlaRS) move 30 to 40 Å to contact the elbow region of their respective tRNAs (fig. S4, B and C) (*27*, *33*). Notably, the largest movements in these aaRSs mainly occur with small accessory domains, which play subsidiary roles in tRNA binding (such as the Ins3 and C-Ala domains). In contrast, the ABD of (αβ)_2_ GlyRS plays a central role in tRNA recognition, and it, together with the HD domain, constitutes almost half of the β subunit. Thus, bacterial (αβ)_2_ GlyRS might be the only aaRS member where a central tRNA recognition domain undergoes such a large spatial movement to capture its tRNA substrate.

Moreover, rotation of the HD domain upon tRNA^Gly^ binding also resulted in a much larger interface between the HD domain and the α subunit [608 Å^2^ versus 252 Å^2^ as calculated by PISA (www.pdbe.org/pisa/)] (fig. S3C) (*34*). The α and β subunits are easy to dissociate, and, as previously discussed, the two subunits fuse via a linker into a single polypeptide in some (αβ)_2_ GlyRSs (*35*). Thus, tRNA binding may facilitate the stabilization of the orphan (αβ)_2_ heterotetramer for catalysis.

### HD domain binds to the major groove of the tRNA^Gly^ acceptor stem

**Fig. 2.**
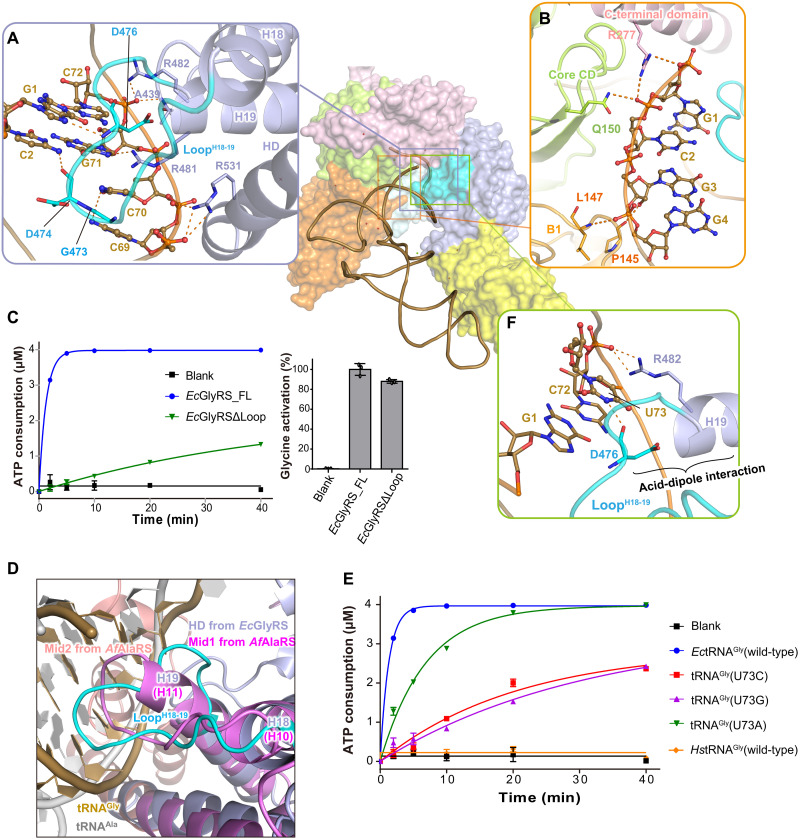
The HD domain of the β subunit of orphan GlyRS participates in the recognition of tRNA^Gly^ acceptor stem and discriminator U73. (**A**) The acceptor stem of tRNA^Gly^ is mainly recognized by the α subunit and the HD domain of the β subunit. The loop (colored in cyan) between helices H18 and H19 (Loop^H18-19^) inserts into the major groove of tRNA^Gly^ acceptor stem and forms broad interactions with the first four base pairs of the acceptor stem. (**B**) The α subunit and the B1 domain of β subunit assist the binding of tRNA^Gly^ acceptor stem through backbone-mediated nonspecific interactions. (**C**) Deletion of the Loop^H18-19^ of orphan *Ec*GlyRS markedly reduced the tRNA-dependent ATP consumption but not the tRNA-independent glycine activation, supporting an important role of Loop^H18-19^ in tRNA^Gly^ binding as observed in the cocrystal structure. The enzymatic assay data here and later are shown as means ± SD (*n* = 3). (**D**) Comparison of orphan *Ec*GlyRS with *Archaeoglobus fulgidus* AlaRS (*Af*AlaRS) (PDB code 3wqy) for their similar modes on recognizing tRNA acceptor stems. Mid1 (purple) and Mid2 (salmon) subdomains of *Af*AlaRS and substrate tRNA (gray) are presented as cartoon. (**E**) The aminoacylation of *E coli* tRNA^Gly^, its U73 mutants, and human tRNA^Gly^ by orphan *Ec*GlyRS. (**F**) Recognition of the discriminator base U73 of tRNA^Gly^ by orphan *Ec*GlyRS. The polar interactions between the first four base pairs and U73 of tRNA^Gly^ and the key residues of *Ec*GlyRS are described as orange dashed lines.

Binding of the acceptor stem of tRNA^Gly^ mainly involves the α subunit and the HD domain of the β subunit. Particularly, the loop between helices H18 and H19 (Loop^H18-19^) and the N-terminal part of H19 from the HD domain insert into the major groove of the tRNA^Gly^ acceptor stem and thereby interact extensively with the first four base pairs (G1∙C72, C2∙G71, G3∙C70, and G4∙C69) (Fig. 2A and fig. S2B) of that stem. The backbones of Gly^473^, Asp^474^, and Asp^476^ from Loop^H18-19^ form four base-specific hydrogen-bonding interactions with G1, C2, C69, and C70, while Arg^481^ at the N terminus of H19 forms a hydrogen bond (H-bond) with the base of G71 (Fig. 2A). In addition to base-specific interactions, the backbones of the first four base pairs form multiple interactions with Ala^439^, Arg^482^, and Arg^531^ from the HD domain of the β subunit (Fig. 2A), with Pro^145^ and Leu^147^ from the B1 domain of the β subunit, and with Gln^150^ and Arg^277^ from the α subunit (Fig. 2B).

Substitution of the first base pair by other nucleotides has been reported to cause an 11- to 43-fold decrease in tRNA^Gly^ aminoacylation (*36*). Replacement of the second base pair lowered aminoacylation by 5- to 10-fold, and mutation of the third base pair resulted in a smaller but still notable decrease (one- to fourfold) (*36*). Thus, these biochemical data confirmed the structural observation that the first four base pairs are important determinants of tRNA^Gly^ recognition. Consistently, sequence analysis using the tRNA^viz^ program (*37*) showed that the first four base pairs are highly conserved among the bacterial tRNA^Gly^ molecules (fig. S5). On the other side, the key residues on Loop^H18-19^ and helix H19 of the HD domain that is involved in interacting with the first four base pairs of tRNA^Gly^ are also highly conserved among the aligned (αβ)_2_ GlyRSs (fig. S6). In addition, in *Ec*GlyRS, deletion of Loop^H18-19^ largely decreased its activity on the tRNA^Gly^-dependent step of aminoacylation as shown in the adenosine 5′-triphosphate (ATP) consumption assay but not glycine activation (Fig. 2C).

In many class II aaRSs, protein sequences outside the aminoacylation domain (e.g., insertion domains or editing domain) facilitate recognition of the acceptor stems and the 3′ termini of their cognate tRNAs (fig. S7). For example, in *Hs*GlyRS, the Ins1 and WHEP domains contact the minor groove of the acceptor stem and 3′ terminus of tRNA, respectively (fig. S7A) (*38*). The N2 domain (editing domain) of threonyl-tRNA synthetase (ThrRS) contacts the minor groove of the acceptor stem (fig. S7B) (*28*). In addition, the insertion domains of histidyl-tRNA synthetase (HisRS) and aspartyl-tRNA synthetase (AspRS) participate in recognizing the 3′ termini of their cognate tRNAs (fig. S5, C and D) (*39*, *40*). In our analysis, AlaRS shows the highest similarity with orphan (αβ)_2_ GlyRS on how the tRNA acceptor stem is recognized (Fig. 2D). While its Mid2 subdomain clamps tRNA from the minor groove side of the tRNA^Ala^ acceptor stem, AlaRS inserts a loop between helices H10 and H11 (Loop^H10-11^) from the Mid1 subdomain into the major groove of the acceptor stem, forming multiple interactions with the bases and backbones of the first three base pairs of tRNA^Ala^ (Fig. 2D and fig. S7E) (*33*). The similar tRNA acceptor stem binding mode between Loop^H10-11^ of the Mid1 subdomain of AlaRS and Loop^H18-19^ of the HD domain of (αβ)_2_ GlyRS (Fig. 2D and fig. S7, E and F) supports the potential evolutionary relationship between these two aaRSs, which has been discussed by others (*12*, *17*, *41*).

### HD domain directly contacts the discriminator base U73

Orphan (αβ)_2_ GlyRS was reported not to glycylate eukaryotic tRNA^Gly^ (Fig. 2E) (*23*). U73 is absolutely conserved in prokaryotic tRNA^Gly^ and as A73 in eukaryotic tRNA^Gly^ (Fig. 1A and fig. S5). Nucleotide 73 is considered as the major determinant for kingdom-specific glycylation of tRNA^Gly^ (*23*, *42*). Our ATP consumption assay confirmed that U73A substitution decreased the aminoacylation of *Ec*tRNA^Gly^ by *Ec*GlyRS and that the U73C and U73G mutants decreased aminoacylation even more strongly than U73A (Fig. 2E).

In our structure of the *Ec*GlyRS∙GlySA∙tRNA^Gly^ ternary complex, the pyrimidine ring of U73 points inward and forms stacks with C72, while Arg^482^ from helix H19 of the HD domain interacts with the backbone phosphate of U73 (Fig. 2F). Because of the insertion of Loop^H18-19^ into the major groove of the acceptor stem, the main chain oxygen of Asp^476^ can form a base-specific H-bonding interaction with N3 of the U73 pyrimidine ring (Fig. 2F). Notably, the local conformation of Loop^H18-19^ is well stabilized by an intramolecular acid–α helix dipole interaction between the β-carboxyl of the conserved Asp^476^ and the N terminus of helix H19 (Fig. 2F and fig. S6). Thus, substitution of U73 by other nucleotides would result in loss of the Asp^476^ H-bond, and, with larger purine bases, such a substitution might also cause a clash with Loop^H18-19^.

The preference for U73 in bacterial tRNA^Gly^ may also have other reasons beyond GlyRS recognition. For instance, bacterial d-aminoacyl-tRNA deacylase (DTD) prefers A73 to U73 (*43*). Thus, the cognate bacterial Gly-tRNA^Gly^ (U73) could escape from unexpected deaminoacylation by DTD, while the noncognate Gly-tRNA^Ala^ (A73) could be efficiently cleaved. Similarly, eukaryotic DTD prefers U73, so that the cognate eukaryotic Gly-tRNA^Gly^ (A73) can be avoided (*43*).

### tRNA^Gly^ CCA end binds to the active site cavity of orphan GlyRS at the aminoacylation mimicking state

**Fig. 3.**
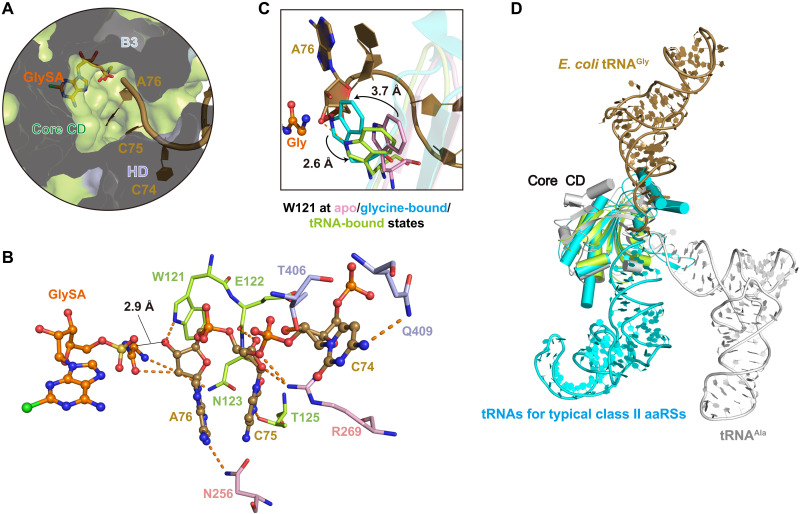
tRNA^Gly^ CCAend binds to orphan *Ec*GlyRS active site cavity at the aminoacylation mimicking state. (**A**) The 3′ CCA end of tRNA^Gly^ extends into the deep aminoacylation pocket of orphan *Ec*GlyRS. *Ec*GlyRS, GlySA and substrate tRNA^Gly^ are presented as surface, sticks, and cartoons, respectively. (**B**) The interactions of CCA end of tRNA^Gly^ with orphan *Ec*GlyRS and intermediate product analog GlySA. The polar interactions are described as orange dashed lines. The distance between the carbonyl group of glycine moiety of GlySA and the 3′-OH of the ribose moiety of A76 is about 2.9 Å. (**C**) Structural movements of Trp121 on the ɑ subunit upon substrate binding. Trp^121^ at apo state [α subunit of *Campylobacter jejuni* GlyRS (*Cj*GlyRS); PDB code 3rf1], glycine-bound state (*Ec*GlyRS·glycine·AMPPNP complex; PDB code 7eiv), and tRNA-bound state are colored in pink, cyan, and green, respectively. (**D**) Structural superimposition of aminoacyl-tRNA synthetases (aaRSs)–tRNA complexes according to their core catalytic domain reveals that *E. coli* tRNA^Gly^ and tRNA^Ala^ locate at positions, which is different from tRNA substrates of other typical class II synthetases including human GlyRS. *E. coli* tRNA^Gly^, tRNA^Ala^, and a representative tRNA for typical class II synthetases [*Hs*GlyRS (PDB code 5e6m), ThrRS (PDB code 1qf6), HisRS (PDB code 4rdx), ProRS (PDB code 1h4s), SerRS (PDB code 1ser), AspRS (PDB code 1c0a), and phenylalanyl-tRNA synthetase (PheRS; PDB code 1eiy)] are colored in dark yellow, gray, and cyan, respectively.

The active cleft on the α subunit is partially covered by the B1, B3, and HD domains of the β subunit, resulting in an active site cavity deeper than that of other typical class II aaRSs (*12*). Therefore, the 3′ CCA end of tRNA^Gly^ adopts an extended conformation to reach the glycyl group of the intermediate product glycyl adenylate (Fig. 3A and fig. S2, D and E), and this conformation of the CCA end was stabilized by forming extensive interactions with residues from the α subunit and HD domain of the β subunit (Fig. 3A).

The cytosine group of C74 is oriented toward the HD domain of the *Ec*GlyRS β subunit and forms an H-bond with the side chain of Gln^409^ (Fig. 3, A and B). In addition, the sugar of C74 interacts with Thr^406^ from the HD domain of the β subunit (Fig. 3B). The cytosine ring of C75 H-bonds with the side chain of Thr^125^ from the α subunit, and the backbone of C75 contacts the side chain of Arg^269^ and the main chain of Glu^122^ from the α subunit (Fig. 3B). The adenine ring of A76 H-bonds with the side chain of Asn^256^ from the α subunit, and its sugar ring also H-bonds with the side chain of Trp^121^ from the α subunit (Fig. 3B).

Notably, structural movement of Trp^121^ on the α subunit was observed upon substrate binding. Compared with GlyRS in the apo state (PDB code 3rf1), the binding of glycine and an ATP analog caused a 3.7-Å movement of Trp^121^ to coordinate glycine by forming a cation-π interaction (*12*). With tRNA^Gly^ bound, Trp^121^ moves back about 2.6 Å to allow A76 to approach the glycyl moiety of the intermediate analog (Fig. 3C). In this state, the 2′-OH of A76 sugar forms two H-bonds with the carbonyl oxygen and amine group of GlySA, respectively (Fig. 3B). The distance between the carbonyl group of glycine moiety and the 3′-OH of A76 is about 2.9 Å (Fig. 3B), suggesting that our complex structure mimics the aminoacylation state in that the 3′-OH of nucleotide A76 of tRNA^Gly^ is positioned to receive the glycyl moiety from the intermediate product, consistent with past work that class II aaRSs fuse the amino acid to the 3′ position (*44*). Except for Thr^406^ in the HD domain (interacts with tRNA^Gly^ CCA end through its backbone) and Thr^125^ in the α subunit (changed to Ser in some GlyRSs), all other residues mentioned above for CCA end interactions are absolutely conserved in the aligned (αβ)_2_ GlyRSs (figs. S6 and S8).

Although orphan (αβ)_2_ GlyRS has some structural similarity to other class II aaRSs on their core catalytic domains, their substrate tRNAs extend their CCA ends to the active site cavities of the cognate aaRSs from different directions (Fig. 3D). When the core catalytic domains of orphan *Ec*GlyRS and other class II aaRSs were superimposed, tRNA^Gly^ located at the position opposite that of most other tRNAs, with only tRNA^Ala^ located between tRNA^Gly^ and other tRNAs (Fig. 3D). In typical class II aaRSs, including eukaryotic GlyRSs, class II signature motif 2 contributes to stabilizing the 3′ CCA end of tRNA by forming charge and hydrogen bonding interactions (fig. S9A). However, this stabilization effect was observed in neither *Ec*GlyRS nor AlaRS (PDB code 3wqy) because of their different modes of tRNA binding (fig. S9B) (*33*, *45*).

### Orphan GlyRS has a unique anticodon binding mode

**Fig. 4.**
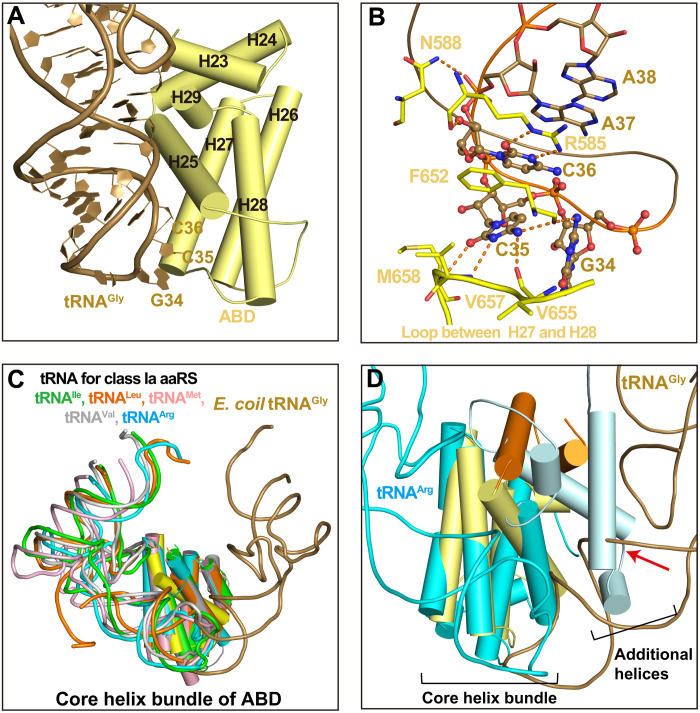
The recognition mode of tRNA anticodon loop by orphan (αβ)_2_ GlyRS. (**A**) The ABD of *Ec*GlyRS, consisting of a core four-helix bundle and a helix-loop-helix motif, approaches the anticodon loop from minor groove side of *E. coli* tRNA^Gly^ anticodon stem. (**B**) A zoomed-in view for anticodon-ABD interactions. The anticodon triplets (G34, C35, and C36) are presented as a ball-and-stick model, while other nucleotides and *Ec*GlyRS residues are shown as sticks. (**C**) Superimposition of the ABD’s core helix bundle of *Ec*GlyRS (yellow) with that of class Ia aaRSs [IleRS (PDB code 1ffy), LeuRS (PDB code 4aq7), MetRS (PDB code 2ct8), ValRS (PDB code 1gax), and ArgRS (PDB code 1f7u)] showed that tRNA^Gly^ binds to the ABD through a direction opposite to other tRNAs. For clarity, only ABDs’ core helix bundles and substrate tRNAs of the aaRS-tRNA complexes are shown. (**D**) With the unique binding mode of tRNA^Gly^, its anticodon stem would cause clashes (indicated with a red arrow) with the accessory helices additional to the core helix bundle of ArgRS’s ABD.

Three anticodons (with G, C, or U at the variable position 34 and C at positions 35 and 36) were used to pair the four glycine codons during protein translation (*46*). In the structure of the orphan *Ec*GlyRS∙GlySA∙tRNA^Gly^ ternary complex, the ABD of the β subunit folds into an all-α structure. The ABD consists of a core four-helix bundle and an additional helix-loop-helix motif, and it specifically recognizes the anticodon triplet of tRNA^Gly^ from the minor groove side of the anticodon stem (Fig. 4A). The G34 of tRNA^Gly^ (GCC) contributes only one H-bond with the backbone of Phe^652^ (Fig. 4B). In contrast, C35 is surrounded by residues mainly from the loop between helices H27 and H28 (Loop^H27-28^), and its cytosine ring forms an H-bonding interaction network with the backbones of Phe^652^, Val^655^, Val^657^, and Met^658^. These four residues are highly conserved in aligned bacterial (αβ)_2_ GlyRSs (Fig. 4B and fig. S6). Furthermore, C36 forms three H-bonds with the conserved Arg^585^ through its cytosine ring and ribose (Fig. 4B and fig. S6).

These observations suggest that C35 plays a vital role in recognizing tRNA^Gly^ by bacterial GlyRSs. Consistently, a C35 mutant of *E. coli* tRNA^Gly^ was reported to reduce charging activity by 105- to 1500-fold, while substitution of C36 by other nucleotides damaged activity by 21- to 41-fold (*36*, *47*). In addition to the anticodon triplet, the phosphate backbone of A37 also contributes to an interaction with Asn^588^. It is worth noting that the stacking interactions among nucleotides C36, A37, and A38 may stabilize the conformation of the tRNA anticodon loop (Fig. 4B).

AaRSs in different subclasses have structurally distinct ABDs. Class IIa aaRSs [ThrRS, HisRS, prolyl-tRNA synthetase (ProRS), and eukaryotic GlyRS] use an α/β-fold ABD at their C termini (*27*, *28*, *40*, *48*). The all-α structure ABD of *Ec*GlyRS is unique to class IIa aaRSs but, interestingly, similar to that of class Ia aaRSs (*17*, *25*). We superimposed the ABD of *Ec*GlyRS with that of the tRNA complex structures of arginyl-tRNA synthetase (ArgRS; PDB code 1f7u), valyl-tRNA synthetase (ValRS; PDB code 1gax), methionyl-tRNA synthetase (MetRS; PDB code 2ct8), LeuRS (PDB code 4aq7), and IleRS (PDB code 1ffy) (*22*, *49*–*52*). *Ec*GlyRS ABD overlaid well with the ABDs of these class Ia aaRSs, particularly for their core four-helix bundles (Fig. 4C). However, the anticodon loops of the different tRNAs approached the anticodon binding site (located at the distal end of the core four-helix bundles) from different sides (Fig. 4C). We took ArgRS as an example, whose ABD showed the highest sequence and structural similarity to that of orphan (αβ)_2_ GlyRS (*17*, *25*). If tRNA^Gly^ binds to the ArgRS ABD as it does in the *Ec*GlyRS∙GlySA∙tRNA^Gly^ ternary complex, then the α helices additional to the core four-helix bundles in the ABD of ArgRS would cause strong clashes with the anticodon stem of tRNA^Gly^ (Fig. 4D). Thus, although orphan (αβ)_2_ GlyRS has a class Ia–like ABD, its tRNA recognition mode is very different from that of class Ia aaRSs.

### The acceptor stem–binding mode of orphan GlyRS further supports the origin of aaRS classifications

**Fig. 5.**
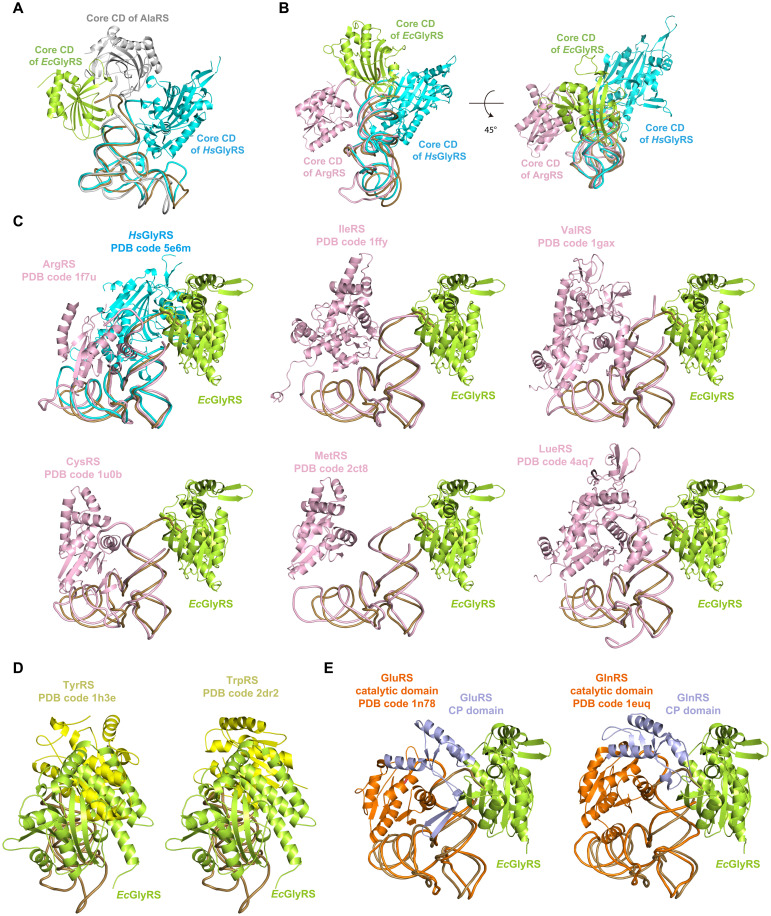
The unusual tRNA binding mode of orphan (αβ)_2_ GlyRS supports subclass-specific pairwise docking of aaRSs on tRNA acceptor stems. (**A**) The catalytic domain of orphan *Ec*GlyRS binds to the acceptor stem of tRNA from a direction different to human GlyRS (a representative class IIa aaRS) and AlaRS [the aaRS member has a special evolutionary relationship with (αβ)_2_ GlyRS] when aligning their tRNA molecules. (**B**) The catalytic domains of most class I aaRSs (such as ArgRS) approach tRNA from the minor groove side of acceptor stem, and the catalytic domains of typical class II aaRSs (such as human GlyRS) bind tRNA acceptor stem from the major groove side. In comparison, the core catalytic domain of orphan *Ec*GlyRS locates between class I aaRSs and typical class II aaRSs and at the top of tRNA acceptor stem. (**C**) The catalytic domain of orphan *Ec*GlyRS could be paired with that of class Ia aaRSs on the acceptor stem of tRNA without clashes. (**D**) The catalytic domain of orphan *Ec*GlyRS would cause clashes with that of class Ic aaRSs when aligning their tRNAs. (**E**) The catalytic domain of orphan *Ec*GlyRS would cause clashes with the insertion sequences (e.g., CP domain) of the catalytic domains of class Ib aaRSs. For clarity, only the α subunit of *Ec*GlyRS (green) and catalytic domains of class Ia (pink), Ib (orange), and Ic (yellow) aaRSs and their respective tRNAs are shown. CP domains of class Ib synthetases are colored in light blue.

While the catalytic domain of most class I aaRSs approaches tRNA from the minor groove side of the acceptor stem, the catalytic domain of typical class II aaRSs binds the tRNA acceptor stem from the major groove side (*9*, *10*). We aligned the tRNAs in *Ec*GlyRS∙GlySA∙tRNA^Gly^ complex and some representative aaRS-tRNA complexes, and the catalytic domain of *Ec*GlyRS was found to bind to the acceptor stem of tRNA from a direction substantially different from other aaRSs, such as *Hs*GlyRS (a representative typical class IIa aaRSs) and AlaRS (has a special relationship with orphan GlyRS) (Fig. 5A). The position of the core catalytic domain of *Ec*GlyRS is between class I and typical class II aaRSs (Fig. 5B).

The catalytic domains of aaRSs in the same subclasses but different classes can be paired without clashes in a highly specific way (Ia paired with IIa, Ib paired with IIb, and Ic paired with IIc), which probably protected the acceptor stems from degradation in the early evolution of life forms (*10*). The cocrystal structure is a bit different from the docking mode of the *Ec*GlyRS-tRNA complex that we proposed previously, where the enzyme bound from the side of the stem (*12*). This difference is due to the unforeseen structural movements and rotations. In the present structure, the orphan GlyRS catalytic domain is at the top of the tRNA acceptor stem (Fig. 5B). When tRNA molecules were aligned, structure superposition revealed that the catalytic domain of orphan *Ec*GlyRS could still fit onto the acceptor stem without clashes with the catalytic domain of class Ia aaRSs (Fig. 5C) but not with the catalytic domain of class Ic aaRSs (Fig. 5D) and the inserted connecting peptide (CP) in the catalytic domain of class Ib aaRSs, which is responsible for the recognition of tRNA acceptor stem and is called “acceptor binding domain” (Fig. 5E) (*53*). Thus, the idiosyncratic structure of the complex provided a fresh test of the “origin of classes” hypothesis, showing that, regardless of its unusual overall structure and tRNA binding mode, orphan (αβ)_2_ GlyRS could still be paired with class Ia aaRSs. This result further supports the hypothesis that the two classes of aaRSs can be defined by how they copair on tRNA.

### Structure of orphan (αβ)_2_ bacterial GlyRS suggests a unique approach to aaRS antibiotics

As 20 essential enzymes, aaRSs have long been considered as ideal targets for developing antibiotics (*20*). The unusual orphan (αβ)_2_ infectious bacterial GlyRS suggested an opportunity to exploit that well-differentiated structure (from host GlyRS and all other aaRSs) for antibiotic development. Infectious bacterial organisms that have orphan (αβ)_2_ GlyRSs include *Streptococcus pneumoniae*, *Helicobacter pylori*, *Chlamydia trachomatis*, *Pseudomonas aeruginosa*, among others.

**Fig. 6.**
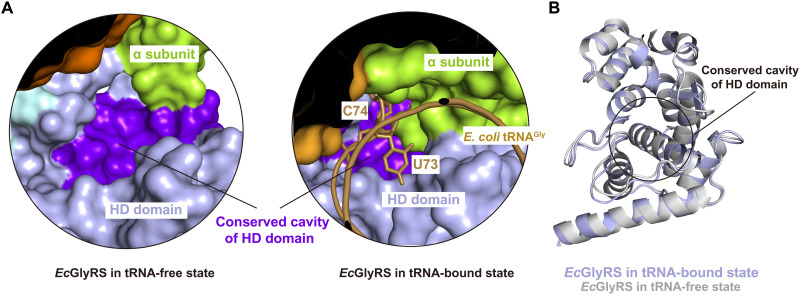
The conserved cavity on HD domain is largely covered by the ɑ subunit and nucleotides U73 and C74 of tRNA acceptor arm after tRNA binding. (**A**) The rotation of the C-terminal part of β subunit upon tRNA binding makes the conserved cavity on HD domain, which is exposed to surface in tRNA-free state, largely covered by α subunit and nucleotides U73 and C74 of the tRNA acceptor arm. (**B**) The structure of HD domain and its conserved cavity have almost no changes upon tRNA binding.

Our previous work identified a highly conserved cavity on the HD domain of orphan GlyRSs, which is absent in eukaryotic GlyRSs. Mutation of residues Lys^364^, Arg^367^, Lys^394^, and Asp^459^ around this bacterial-specific cavity reduced the aminoacylation activity to bacterial tRNA^Gly^, but the mechanism was unclear (*12*). tRNA binding requires a large rotation of the C-terminal HD and ABD domains of the β subunit. While this cavity on HD is exposed in the structure of the tRNA-free state enzyme (Fig. 6A), it gets largely covered by the ɑ subunit and closely contacts nucleotides U73 and C74 of the acceptor arm of bound tRNA^Gly^ in the *Ec*GlyRS∙GlySA∙tRNA^Gly^ complex (Fig. 6A). However, the structure itself of this cavity is not reshaped by this rotation (Fig. 6B). Likely, chemicals binding to this cavity could disrupt the 3′ CCA end of bacterial tRNA^Gly^ from accessing the active site. Thus, this conserved cavity on the HD domain, which is idiosyncratic to the orphan GlyRSs, presents a unique opportunity to go beyond the highly conserved active sites to find and develop antibiotics.

## DISCUSSION

Eukaryotic GlyRS, like class IIa ThrRS, HisRS, and ProRS, has an α/β-fold ABD at the C terminus and recognizes the anticodon triplet of tRNA^Gly^ from the major groove side of the anticodon stem (*38*). In contrast, the ABD in orphan (αβ)_2_ GlyRS is an all-α structure, and it binds anticodon triplets from the minor groove side of the stem (Fig. 4A). Studies have suggested that orphan GlyRS is related to the ancestor of AlaRS (*12*, *41*), a special class IIa aaRS that lacks an ABD and instead recognizes tRNA^Ala^ through the G3∙U70 identity base pair (*33*). Possibly, the preorphan GlyRS did not have an ABD and recognized tRNA^Gly^ mainly on the basis of U73 and the first four base pairs. When preorphan GlyRS evolved to more accurately and effectively capture tRNA^Gly^ by integrating an ABD, the ABD was not obtained from other class IIa aaRSs but from ArgRS, because the ABD of ArgRS has multiple specific interactions with C35 of tRNA^Arg^. For other tRNAs with C at position 35, their corresponding aaRSs recognize tRNA either through the shape of the cognate tRNA [e.g., seryl-tRNA synthetase (SerRS) and cysteinyl-tRNA synthetase (CysRS)] or by forming more interactions with another nucleotide in the anticodon loop [e.g., trytophanyl-tRNA synthetase (TrpRS)] (*29*, *54*–*56*).

A minihelix comprising the acceptor-TpsiC stem of tRNA^Gly^ is charged by eukaryotic and prokaryotic GlyRSs (*42*). The first two base pairs of the acceptor stem are well conserved as G1∙C72 and C2∙G71 in tRNA^Gly^ molecules from all three kingdoms of life (fig. S5). Mutations of either base pair strongly reduced the charging activity of bacterial and eukaryotic tRNA^Gly^ by their cognate GlyRSs (*36*). Both *Hs*GlyRS and *Ec*GlyRS have direct base-specific interactions with the first two base pairs of their respective tRNA^Gly^ (Fig. 2A) (*38*). However, different mechanisms are used. For example, for *Hs*GlyRS, Arg^283^ from the catalytic domain forms three H-bonds with G1 and G71 (*38*). In contrast, Asp^474^, Asp^476^, and Arg^481^ from the HD domain of orphan *Ec*GlyRS form three H-bonds with C2, G1, and G71, respectively (Fig. 2A). The use of distinct domains to retain the acceptor stem recognition of the first two base pairs strongly suggests the cross-kingdom evolutionary pressure, which focuses on the same base pairs for a critical discrimination of tRNA^Gly^. However, orphan GlyRS charges only tRNA^Gly^ from bacteria but not tRNA^Gly^ from eukaryotes. N73 (conserved as U in bacteria and A in eukaryotic tRNA^Gly^) is considered as a key discriminator for kingdom specificity of tRNA^Gly^ glycylation. Thus, despite common cross-kingdom recognition of the first two base pairs of the acceptor stem, N73 has ostensibly evolved to block cross-kingdom charging of tRNA^Gly^. However, substitution of U73 by A in *E. coli* tRNA^Gly^ decreased but did not completely abolish the aminoacylation activity in our assay (Fig. 2E), consistent with a previous report that *E. coli* tRNA^Gly^ U73A mutant caused only an 11-fold decrease in *V*_max_/*K*_m_ (*35*). Thus, other discriminators beside U73 may contribute to the kingdom specificity of tRNA^Gly^, which requires further studies.

While traditional aaRS inhibitors are active site–targeted competitors of amino acid and/or ATP binding, other studies have revealed that blocking aaRS-tRNA interactions might be a viable alternative strategy for drugging aaRSs (*57*–*62*). The first representative cocrystal structure of orphan (αβ)_2_ GlyRS in complex with tRNA^Gly^ presented here clarifies the mechanisms of how the unique structure of orphan (αβ)_2_ GlyRS recognizes its substrate tRNA^Gly^. Our structural study not only reinforces the paradigm of the origin of aaRS classifications but also shows bacterial-specific large conformational changes and nonactive site regions associated with the binding of tRNA^Gly^, which informs on a potential approach to the development of bacteria-specific antimicrobials.

## MATERIALS AND METHODS

### Protein expression and purification

The coding sequence for the full-length α [UniProt identifier (ID): P00960] and β (UniProt ID: P00961) subunits of *Ec*GlyRS were amplified from the genomic DNA of *E. coli* strain K12 with polymerase chain reaction (PCR) and inserted into the plasmids pET20b and pET28a, respectively. The β subunit has a hexahistidine tag at the C terminus for protein purification. *E. coli* BL21(DE3) cells cotransformed with two plasmids were grown at 37°C in LB medium supplemented with ampicillin (100 mg/liter) and kanamycin (50 mg/liter) until optical density at 600 nm reached 0.6. Coexpression of the α and β subunits of *Ec*GlyRS was induced by adding 0.2 mM isopropyl-β-d-thiogalactopyranoside. After growth at 20°C for 20 hours, *E. coli* cells were harvested by centrifugation at 5000 revolutions per min (rpm) for 30 min. The cell pellets were resuspended and sonicated in lysis buffer [50 mM tris-HCl (pH 8.0), 400 mM NaCl, and 20 mM imidazole]. The cell lysate was centrifuged at 18,000 rpm for 30 min at 4°C, and the supernatant was loaded onto a Ni–nitrilotriacetic acid (NTA) column preequilibrated with lysis buffer. The Ni-NTA column was washed with 20 column volumes of lysis buffer to remove impurities, and the target protein was eluted with elution buffer [50 mM tris-HCl (pH 8.0), 400 mM NaCl, and 200 mM imidazole]. The protein in elution fraction was concentrated to 15 to 20 mg/ml using a 50-kDa Ultra-15 centrifugal filter device and then further purified using a HiLoad 16/60 Superdex 200 pg column with the running buffer [200 mM NaCl and 20 mM tris-HCl (pH 8.0)]. Protein purity was assessed with SDS–polyacrylamide gel electrophoresis (SDS-PAGE). The purified (αβ)_2_ *Ec*GlyRS protein was concentrated in the storage buffer [100 mM NaCl and 10 mM tris-HCl (pH 7.0)] and frozen at −80°C before use.

### In vitro transcription of tRNA^Gly^

*E. coli* tRNA^Gly^ (GCC), the most favorable tRNA^Gly^ molecule in *E. coli* cells (*63*, *64*), was produced with in vitro T7 polymerase transcription. The initial DNA template was generated by PCR using primer1 (5′-**TAATACGACTCACTATA**GCGGGAATAGCTCAGTTGGTAGAGCACGACCTTGCCAAG-3′) and primer2 (5′-TGGAGCGGGAAACGAGACTCGAACTCGCGACCCCGACCTTGGCAAGGTCGTGCTC-3′). These two primers covered the full sequence of tRNA^Gly^ (GCC) and partially overlapped with each other (underlined nucleotides), and primer1 also contains a T7 promoter sequence (nucleotides in bold). The PCR product was further amplified by the second round of PCR with primer3 (5′-TAATACGACTCACTATAGCGGGAATAGC-3′) and primer4 (5′-*UG*GAGCGGGAAACGAGACTCG-3′), and the product was then used as the DNA template for in vitro T7 transcription assay without additional purification. The first two nucleotides at the 5′ terminus of primer4 (nucleotides in italics) were methylated at their 2′-hydroxyl groups to reduce nontemplated nucleotide addition by the T7 RNA polymerase (*65*). The T7 transcription reaction consisted of 200 mM tris-HCl (pH 8.0), 2 mM spermidine, 10 mM dithiothreitol (DTT), 20 mM MgCl_2_, DNA template (50 ng/μl), 4 mM ATP, 4 mM uridine 5′-triphosphate, 4 mM guanosine 5′-triphosphate, 4 mM cytidine 5′-triphosphate, 20 mM guanosine 5′-monophosphate, and 2 μM T7 polymerase. After incubation at 37°C for 3 to 4 hours, the transcripts were desaturated at 95°C for 5 min. The transcripts were purified by 12% PAGE supplemented with 8 M urea. The target tRNA transcripts were extracted from gel with 0.5 M ammonium acetate, precipitated by ethanol at −20°C overnight, collected by centrifugation, and redissolved in a buffer consisting of 20 mM tris-HCl (pH 8.0) and 1 mM EDTA. The tRNA product was heated at 65°C for 5 min and then refolded by slowly cooling to room temperature after the addition of 10 mM MgCl_2_. The refolded tRNA was concentrated with a 3-kDa Ultra-4 centrifugal filter device (Millipore), and its final concentration was determined by the ultraviolet absorbance at the wavelength of 260 nm. The tRNA product was stored at −80°C before use.

### Crystallography

The sitting-drop vapor diffusion method was used to crystallize the *Ec*GlyRS∙GlySA∙tRNA^Gly^ complex. In brief, the full-length (αβ)_2_ *Ec*GlyRS (22 mg/ml) was mixed with tRNA^Gly^(GCC) (in vitro transcript, 7.5 mg/ml) and 2 mM GlySA. GlySA was synthesized as described in scheme S1. After an incubation for 30 min on ice, crystallization drops were set up by mixing 0.5 μl of protein complex solution with 0.5 μl of reservoir solution [0.15 M MgCl_2_, 0.1 M NaCl, 0.1 M tris-HCl (pH 8.5), 30% polyethylene glycol, molecular weight 300]. Crystallization drops were equilibrated against 60 μl of reservoir solution at 8°C. Large crystals appeared after 4 to 7 days and were directly flash-frozen in liquid nitrogen without additional cryoprotectant solution. Diffraction data were collected using a single crystal at 100 K with a wavelength of 0.979 Å at beamline BL02U1 of the Shanghai Synchrotron Radiation Facility, China. The oscillation angle was 0.5° for each frame, and the whole dataset contained 360 frames.

The diffraction data were automatically processed with Aquarium (*66*), which used the autoPROC (*67*). The structure was solved using the molecular replacement method in the program MOLREP (*68*) with structures of *Ec*GlyRS575∙glycine∙AMPPNP complex (PDB code 7eiv), full-length *Ec*GlyRS β subunit predicted by AlphaFold2 (www.alphafold.ebi.ac.uk/entry/P00961) (*31*), and free *Geobacillus kaustophilus* tRNA^Gly^ (PDB code 4mgm) as search models. Iterative refinement of the structure model was carried out using Coot (*69*) and Refmac5 (*70*). The stereochemical quality of the final model was assessed using MolProbity (*71*). The statistics for the data collection and structural refinements are listed in table S1. The structure was analyzed in PyMOL (www.pymol.org), which was also used to create the figures.

### ATP consumption assay

An ATP consumption assay was used to evaluate the aminoacylation activity of *Ec*GlyRS on tRNA^Gly^ as described (*12*). In brief, the 30 μl of mixture containing 50 nM *Ec*GlyRS, 4 μM ATP, 500 μM glycine, *E. coli* tRNA^Gly^ (GCC) (0.25 mg/ml), 30 mM Hepes (pH 7.5), 150 mM NaCl, 30 mM KCl, 40 mM MgCl_2_, 1 mM DTT, and 0.1% bovine serum albumin was prepared and incubated at room temperature. Five microliters of the mixture was collected from the reaction at different time points (2, 5, 10, 20, and 40 min) and mixed with 5 μl of Kinase-Glo Reagent (Promega) in a 384-well microplate to stop the reaction. After 15 min, the luminescence which reflects the concentration of the remaining ATP in the well was read on a Synergy H1 microplate reader (BioTek). The reaction without adding tRNA^Gly^ was used as the control. Last, the ATP consumption was calculated as the difference between the luminescence in the well with RNA^Gly^ and the well without tRNA^Gly^. Each reaction was repeated three times, and the results are expressed as the means ± SD (*n* = 3). Statistical analyses were performed with GraphPad Prism 6.0 software, and a one-phase association equation was used for the curve fitting of the ATP consumption assay.

### Measurement of glycine activation activity of *Ec*GlyRS

Glycine activation by *Ec*GlyRS_FL and *Ec*GlyRS∆Loop was detected by using a coupled assay (*12*). *Ec*GlyRS could catalyze an adenylyl-imidodiphosphate (AMP-PNP) and a glycine to generate a molecule of glycyl-AMP, which is attacked by a pyrophosphate to form a glycine and one ATP. Hexokinase consumes one ATP to phosphorylate glucose to glucose 6-phosphate, from which glucose 6-phosphate dehydrogenase then consumes one nicotinamide adenine dinucleotide phosphate (NADP^+^) to produce one 6-phosphogluconate and one NADPH (reduced form of NADP^+^). The experiment was conducted at room temperature in a clear 96-well microplate (Corning). Ninety microliters of reaction buffer consisting of 50 mM Hepes (pH 7.5), 10 mM MgCl_2_, 50 mM KCl, 1 mM DTT, 3 mM AMP-PNP, 10 mM glycine, 10 mM d-glucose, 2.5 mM sodium pyrophosphate, 0.5 mM NADP^+^, 2.5 U of yeast hexokinase, and 2.5 U of glucose 6-phosphate dehydrogenase was placed in the wells of the microplate. The reactions were started by adding 10 μl of *Ec*GlyRS_FL or *Ec*GlyRS∆Loop (final enzyme concentrations were 200 nM) to each well. The reduction of NADP^+^ to NADPH in the coupled assay was continuously recorded for the first 10 min by monitoring the absorbance at 340 nm using a Synergy H1 microplate reader (BioTek), and the reactions without adding inorganic pyrophosphate were used as blank controls. The slope of NADPH formation in the first 10 min (equal to the glycine activation rate) of wild-type *Ec*GlyRS_FL was defined as 100%. The results were from three independent assays, and the error bars represent SDs.

### Data analysis and figure preparation

Figures were created using the PyMOL. Protein sequence alignment analysis was performed using Clustal Omega program (*72*). The conservation scores were calculated by program Jalview (*73*). tRNA sequence analysis was performed using the tRNA^viz^ program (*37*). Statistical analysis was performed using GraphPad Prism 6.0 software, each enzymatic activity assay was repeated three times, and the results are expressed as the means ± SD (*n* = 3).

## Supplementary Materials

### This PDF file includes:

Supplementary Method Scheme S1 Figs. S1 to S9 Table S1 References

## Notes

View/request a protocol for this paper from *Bio-protocol*.
