# Supplementary material for: The binding mode of orphan glycyl-tRNA synthetase with tRNA supports the synthetase classification and reveals large domain movements
Source: Sci Adv. 2023 Feb 8;9(6):eadf1027. doi: 10.1126/sciadv.adf1027 (PMC9908026; doi:10.1126/sciadv.adf1027)
Supplement: Supplementary file 1 — Supplementary Method Scheme S1 Figs. S1 to S9 Table S1 References [file sciadv.adf1027_sm.pdf]

Science Advances
AAAS

Supplementary Materials for

The binding mode of orphan glycyl-tRNA synthetase with tRNA supports the synthetase classification and reveals large domain movements

Lu Han *et al.*

Corresponding author: Huihao Zhou, zhuihao@mail.sysu.edu.cn; Paul Schimmel, schimmel@scripps.edu

Sci. Adv. **9**, eadf1027 (2023)
DOI: 10.1126/sciadv.adf1027

This PDF file includes:

Supplementary Method
Scheme S1
Figs. S1 to S9
Table S1
References

Supplementary Method

Synthesis of GlySA

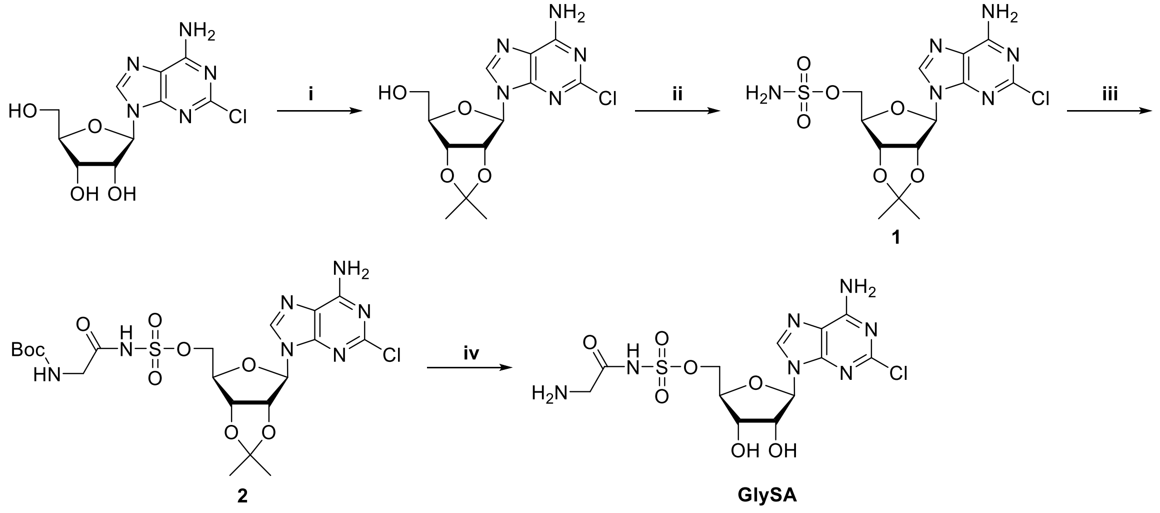

Scheme S1. The synthesis of intermediate analog. (i) p-toluenesulfonic acid, DMOP, DMF, N_2, rt, 20 h; (ii) NH_2SO_2Cl, DMA, N_2, rt, 4h; (iii) Boc-Gly-OSu, DBU, DMF, rt, 8h; (iv) TFA: H_2O=5:1, rt, 2h.

2-*chloro-5'-O-[N-(glycl)sulfamoyl) adenosine* (**GlySA**). **GlySA** (*26*) was prepared as the procedure shown in Scheme S1. The compound [(3aR,4R,6R,6aR)-6-(6-Amino-2-chloro-9Hpurin-9-yl)-2,2-dimethyl-tetrahydro-2H-furo[3,4-d][1,3]-3-dioxol-4-yl]methyl sulfamate (**1**) was prepared via the similar method described previously (*74*) with high yield. The compound **1** (210 mg, 1.0 equiv) was dissolved in 10 mL DMF, and then DBU (84 µL, 1.1 equiv) and Boc-Gly-OSu (150 mg, 1.1 equiv) were added to the reaction mixture. After stirring for 8 h at room temperature, the reaction mixture was diluted with 25 mL brine and extracted with dichloromethane. The organic layers were dried, concentrated and purified by flash chromatography to obtain the compound ((3aR,4R,6R,6aR)-6-(6-amino-2-chloro-9H-purin-9-yl)-2,2-dimethyltetrahydrofuro[3,4-d][1,3]dioxol-4-yl)methyl ((tert-butoxycarbonyl)glycyl)sulfamate (**2**) as a colorless glassy solid. 144 mg compound **2** was dissolved in TFA/H_2O (5:2, 3 mL) and stirred at room temperature for 0.5 h. The reaction mixture was concentrated in vacuo, and then purified by preparative HPLC to afford the final product GlySA as colorless solid. ^{1}H NMR (500 MHz, CD_3OD): δ 8.31 (s, 1H), 6.00 (d, J = 4.3 Hz, 1H), 4.61 – 4.56 (m, 2H), 4.51 (dd, J = 11.2, 4.0 Hz, 1H), 4.39 (t, J = 5.1 Hz, 1H), 4.30 (dd, J = 8.2, 3.9 Hz, 1H), 3.77 (s, 2H). MS (ESI) *m/z*: calcd for $C_{12}H_{17}N_7O_7SCl$ [M + H]$^+$, 438.05, found, 438.05.

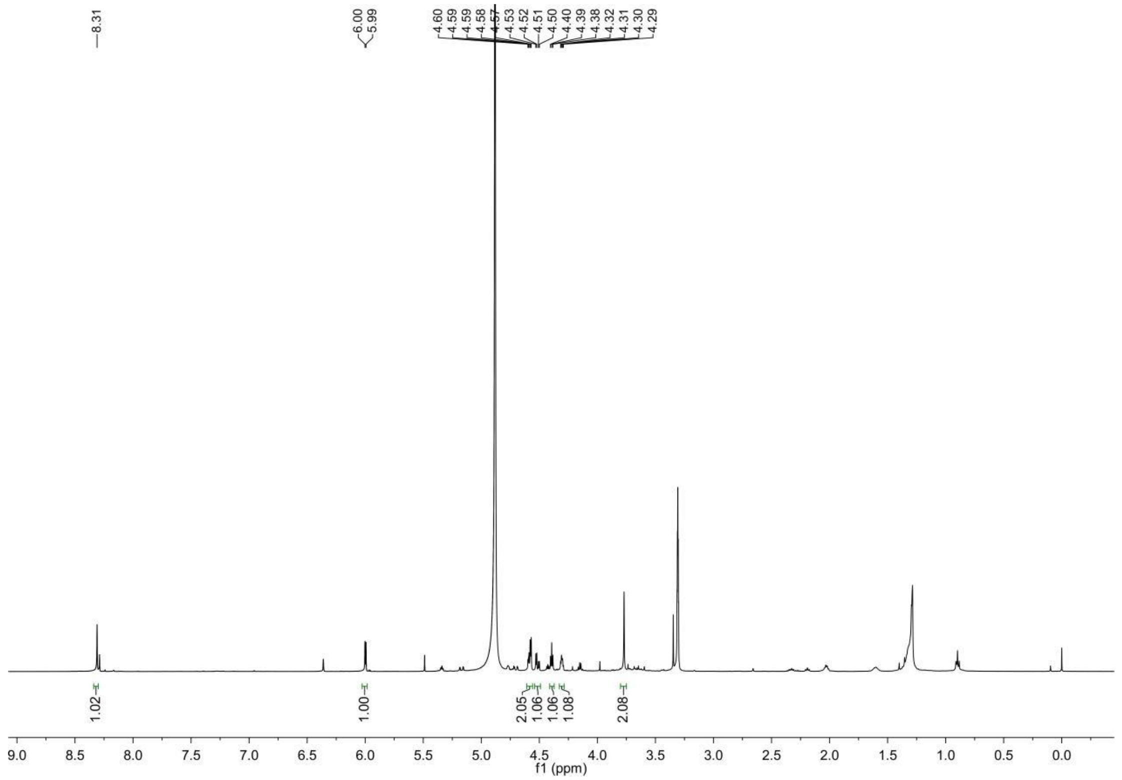

8.31
6.00
5.99
4.60
4.59
4.59
4.58
4.57
4.53
4.52
4.51
4.50
4.40
4.39
4.38
4.32
4.31
4.30
4.29
1.02
1.00
2.05
1.06
1.06
1.08
2.08
9.0 8.5 8.0 7.5 7.0 6.5 6.0 5.5 5.0 4.5 4.0 3.5 3.0 2.5 2.0 1.5 1.0 0.5 0.0
f1 (ppm)

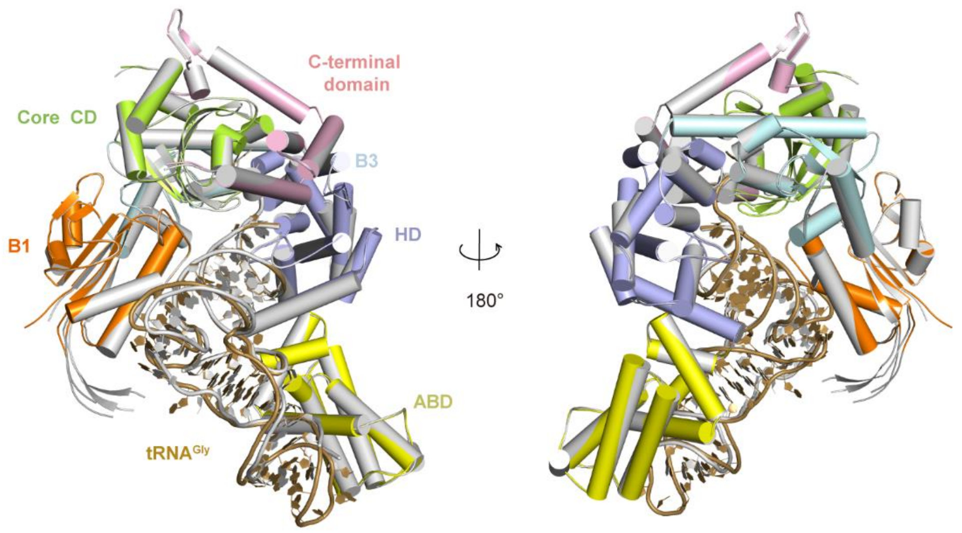

Fig. S1. Structure superimposition of the two protomers of orphan *Ec*GlyRS revealed that both their own structures and their tRNA binding modes are almost identical. One protomer and its substrate tRNA^{Gly} are colored the same as Fig. 1B, while the other protomer and its corresponding tRNA^{Gly} are colored in gray.

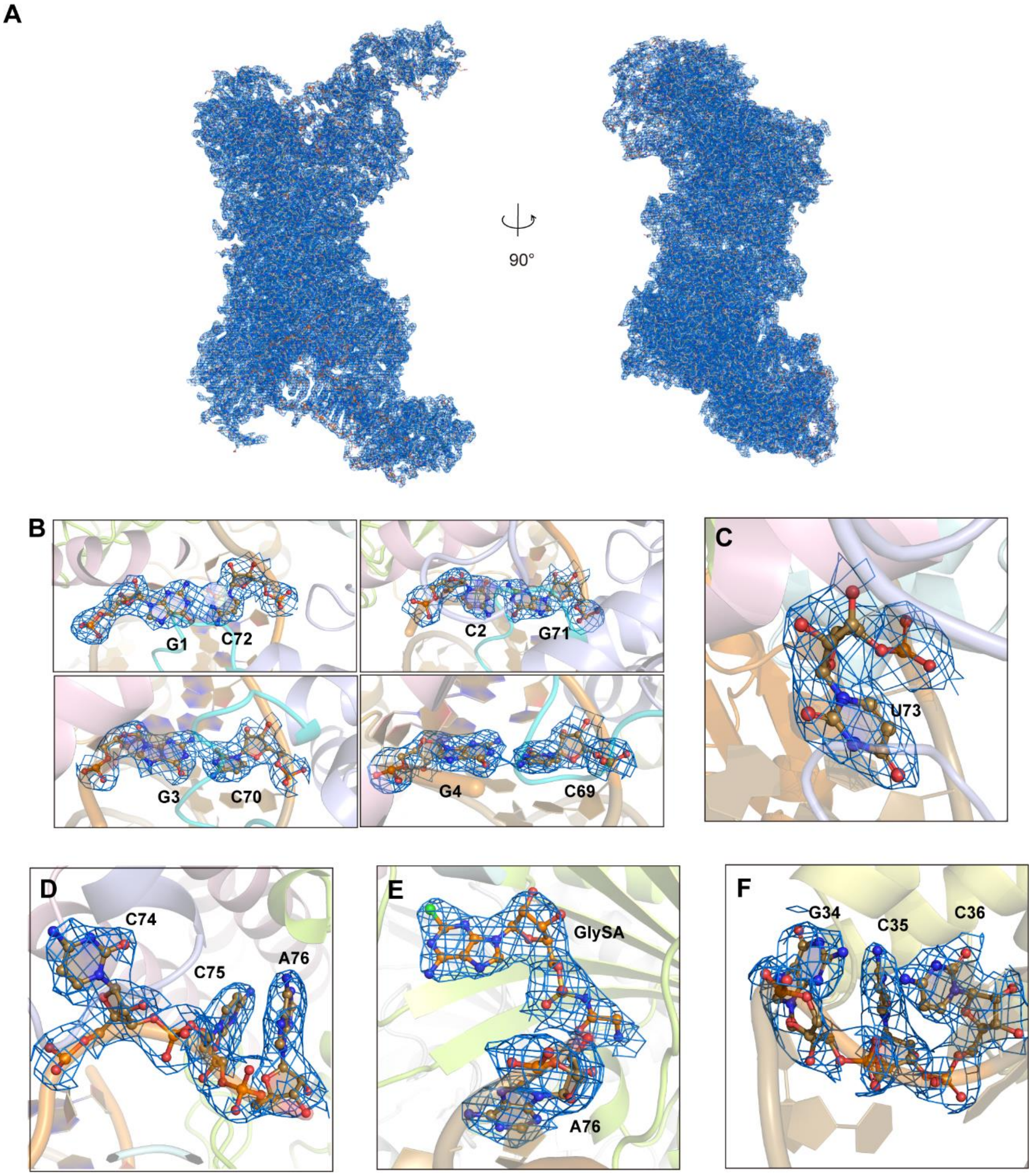

Fig. S2. Electron density map of orphan *Ec*GlyRS·GlySA·tRNA^{Gly} complex structure. (A) 2Fo-Fc omit electron density maps of the protein and nucleic acid chain are drawn as blue meshes contoured at 1.0 σ. **(B-F)** 2Fo-Fc omit electron density maps of the first four base pairs of tRNA^{Gly} acceptor stem **(B)**, U73 **(C)**, the 3' CCA-end **(D)**, GlySA **(E)** and anticodon triplets **(F)** are shown as blue meshes and contoured at 1.0 σ.

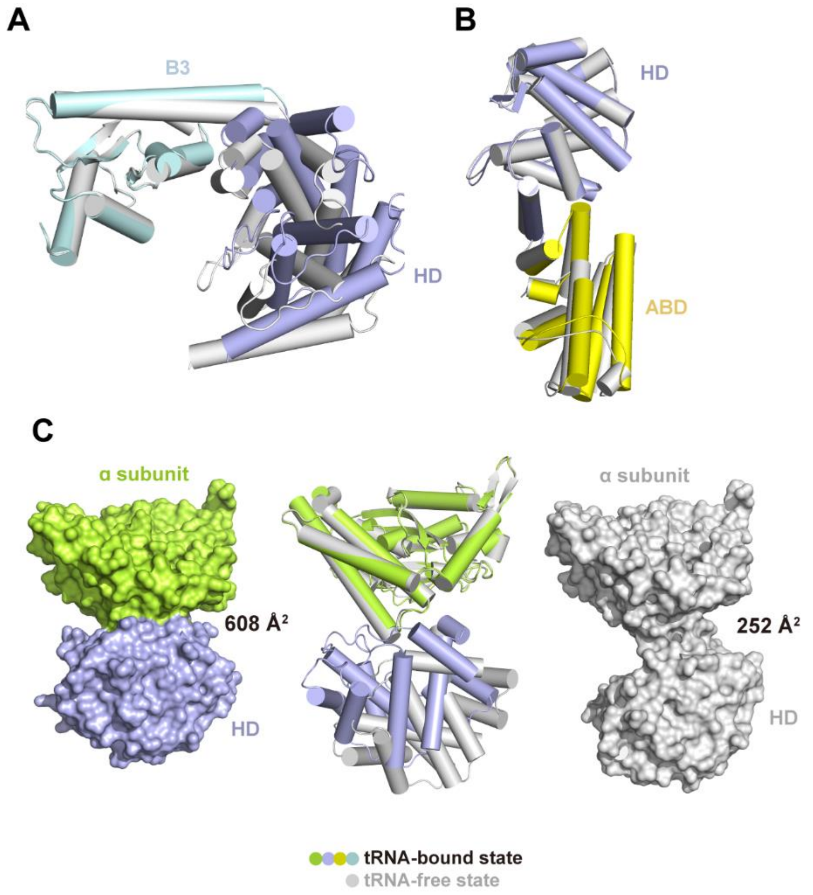

Fig. S3. The HD domain and ABD rotate as a whole upon tRNA^{Gly} binding, which also enlarges the interface between the HD domain and the α subunit. (A) The HD domain in the β subunit of orphan *Ec*GlyRS bound with tRNA rotates related to that of *Ec*GlyRS without tRNA when their B3 domains are aligned. **(B)** The ABD of *Ec*GlyRS bound with tRNA aligns well with that of *Ec*GlyRS without tRNA when their HD domains are superimposed. Thus, rotation of the β subunit C-terminal part mainly happens at the linker between the B3 and HD domains, and HD domain and ABD rotate as a whole. **(C)** When α subunit in tRNA-bound state is superimposed to that in tRNA-free state, the HD domain moves towards α subunit in tRNA-bound state, resulting in a larger interface between HD domain and α subunit. In **(A-C)**, orphan *Ec*GlyRS in tRNA-bound state is colored the same as Fig. 1B, while orphan *Ec*GlyRS in tRNA-free state is colored in gray. The interface areas were calculated using program PISA.

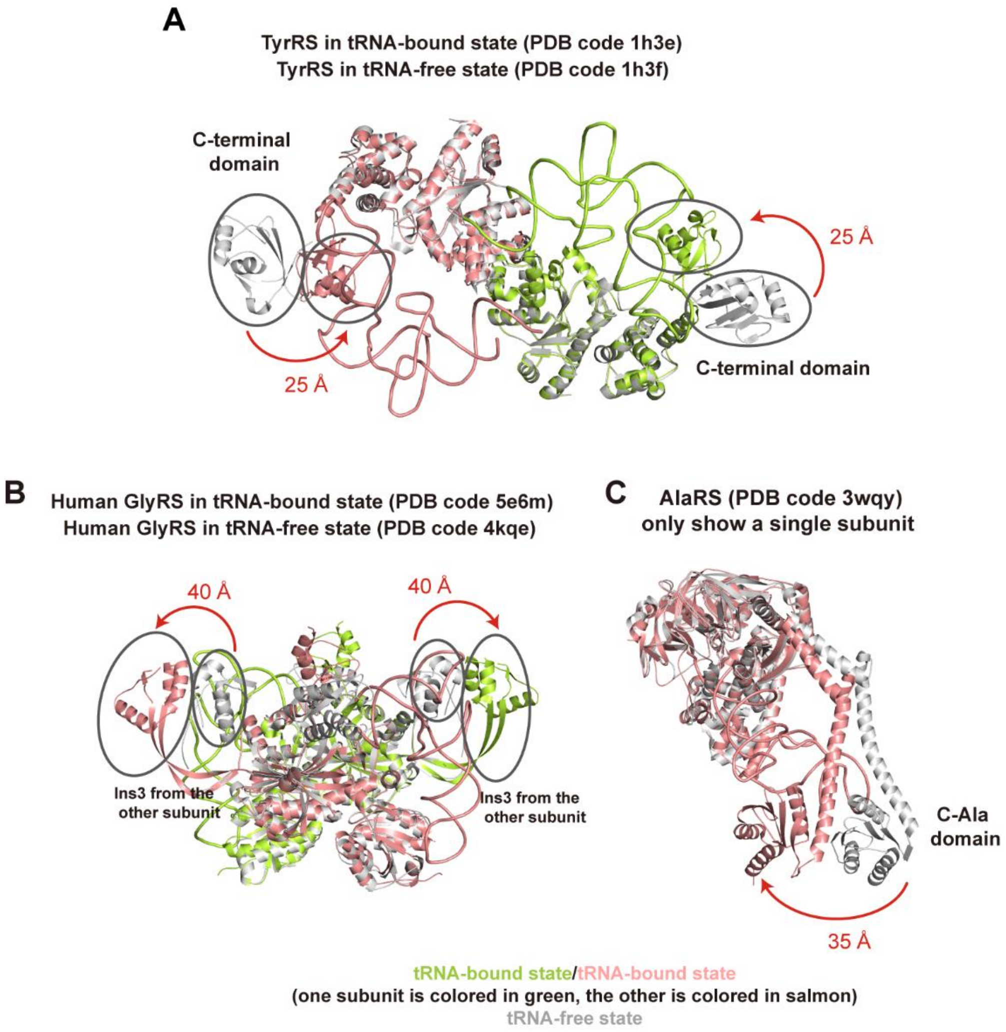

Fig. S4. Large conformational changes of aaRSs upon tRNA binding. **(A)** The largest structural movement of class I aaRSs induced by tRNA binding is the C-terminal domain of TyrRS. It moves about 25 Å to contact and recognize the long variable loop of $tRNA^{Tyr}$. **(B)** In human GlyRS, the Ins3 domain from the other subunit opens up to contact the elbow region of $tRNA^{Gly}$. **(C)** The C-Ala domain of AlaRS moves about 35 Å to contact the elbow region of $tRNA^{Ala}$. In **(A-C)**, aaRSs in tRNA-bound state are colored in green for one subunit and salmon for the other, while that in tRNA-free state are colored in gray.

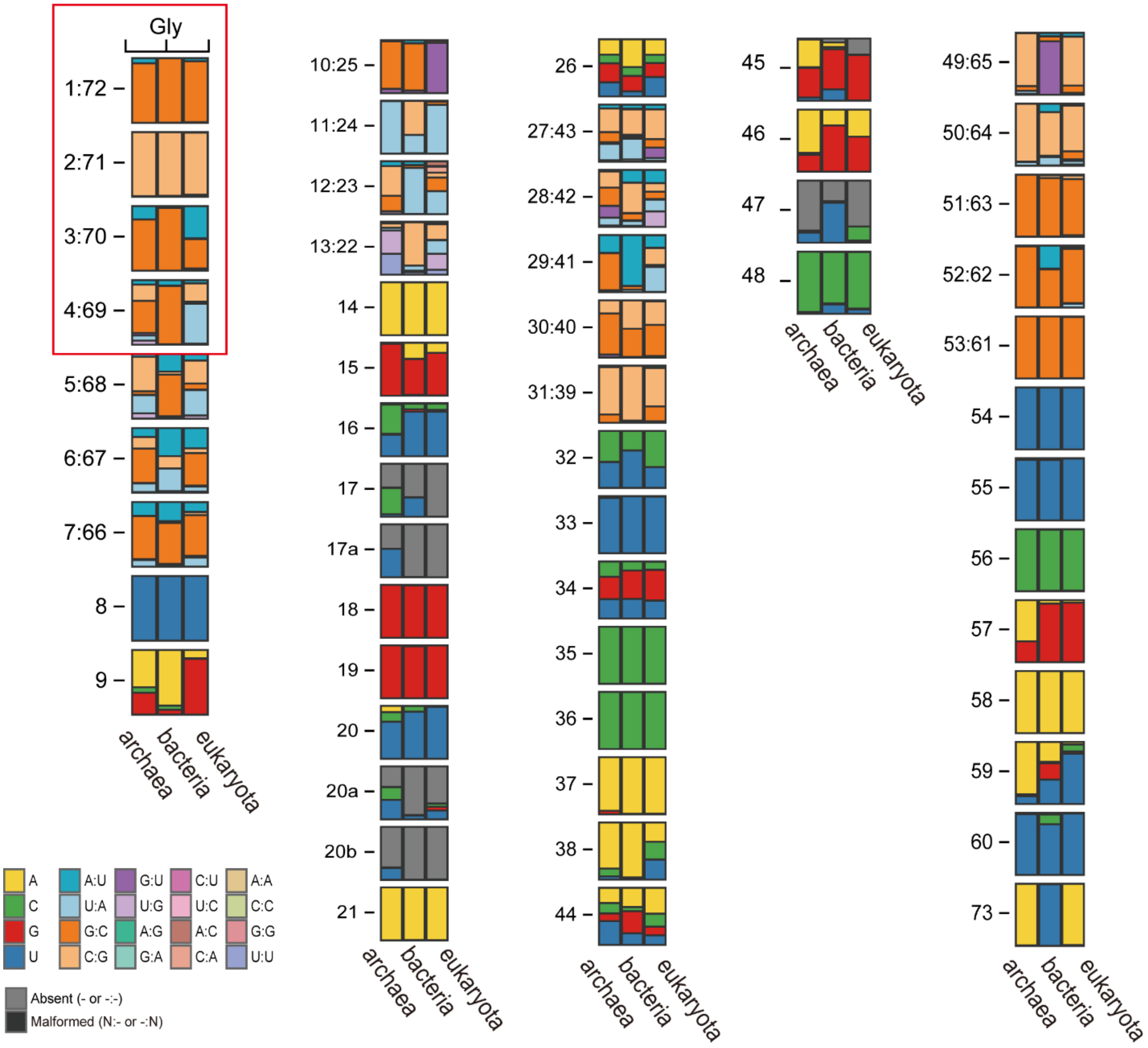

Fig. S5. Sequence analysis of tRNA^{Gly} among archaea, bacteria and eukaryotes using tRNA^{viz} . Sequence feature distribution for all positions of tRNA^{Gly} molecules among three domains of life were analyzed using program tRNA^{viz} (*37*). The first four base pairs of the acceptor stem are squared in red.

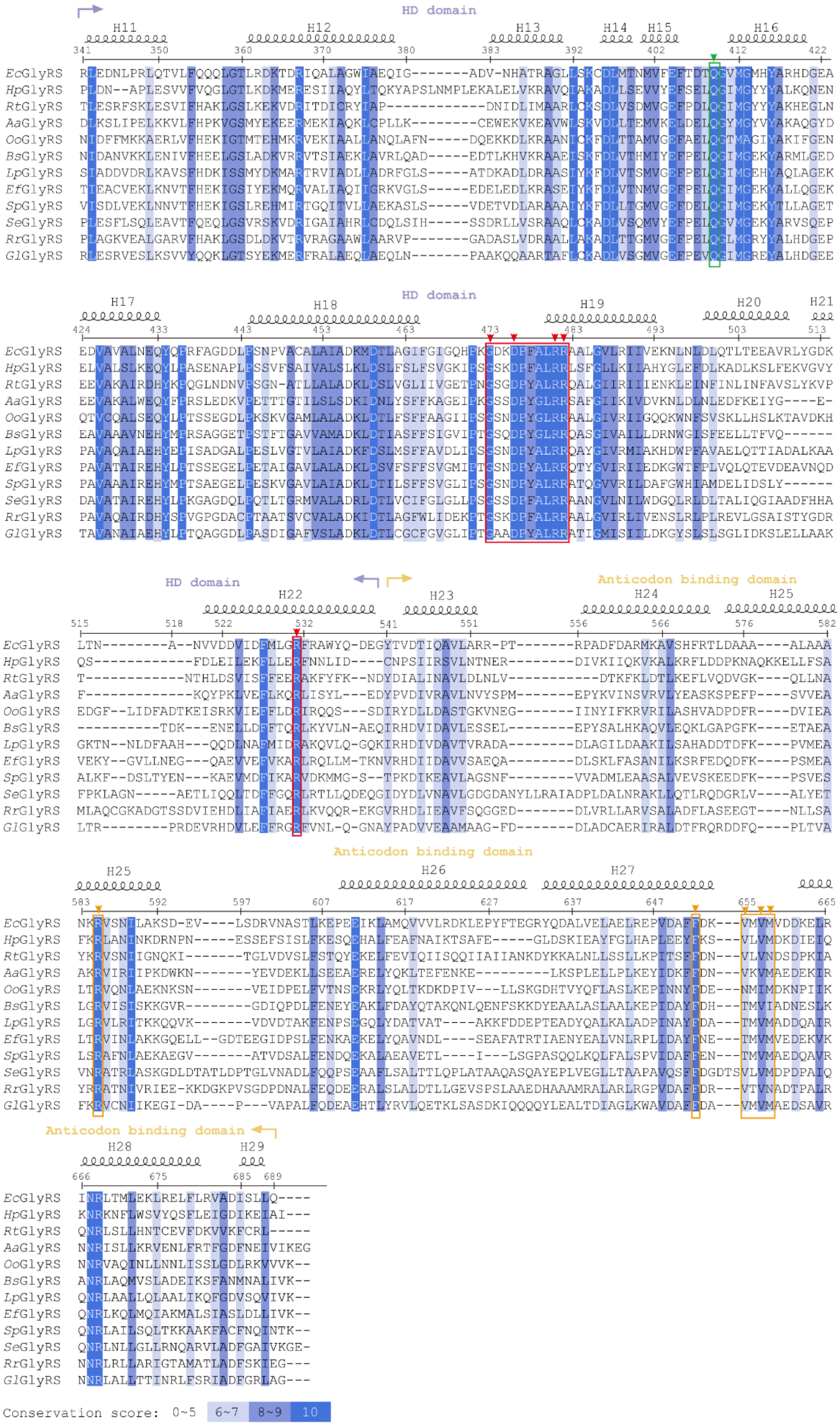
HD domain
H11 H12 H13 H14 H15 H16
H17 H18 H19 H20 H21
H22 H23
Anticodon binding domain
H24 H25 H26 H27 H28 H29
EcGlyRS
HpGlyRS
RtGlyRS
AaGlyRS
OoGlyRS
BsGlyRS
LpGlyRS
EfGlyRS
SpGlyRS
SeGlyRS
RrGlyRS
GlGlyRS
Conservation score: 0~5 6~7 8~9 10

Fig. S6. Sequence alignments of the HD domain and the ABD of β subunits from orphan GlyRSs. Protein sequences of *Escherichia coli* GlyRS (*Ec*GlyRS, UniProtKB ID: P00961), *Helicobacter pylori* GlyRS (*Hp*GlyRS, UniProtKB ID: B5Z7X4), *Rickettsia typhi* GlyRS (*Rt*GlyRS, UniProtKB ID: Q68VR4), *Aquifex aeolicus* GlyRS (*Aa*GlyRS, UniProtKB ID: O67898), *Oenococcus oeni* GlyRS (*Oo*GlyRS, UniProtKB ID: Q04F69), *Bacillus subtilis* GlyRS (*Bs*GlyRS, UniProtKB ID: P54381), *Lacticaseibacillus paracasei* GlyRS (*Lp*GlyRS, UniProtKB ID: Q038U3), *Enterococcus faecalis* GlyRS (*Ef*GlyRS, UniProtKB ID: Q831U3), *Streptococcus pneumoniae* GlyRS (*Sp*GlyRS, UniProtKB ID: B8ZL20), *Synechococcus elongatus* (*Se*GlyRS, UniProtKB ID: Q31SB9), *Rhodospirillum rubrum* (*Rr*GlyRS, UniProtKB ID: Q2RQ43), *Geobacter lovleyi* GlyRS (*Gl*GlyRS, UniProtKB ID: B3E621) were aligned using Clustal Omega program (*72*). The secondary structures corresponding to *Ec*GlyRS are shown above the sequences. The conservation scores were calculated by the program Jalview (*73*) and presented in various shades of blue. Key residues which recognize the acceptor stem, 3' CCA-end and anticodon of $tRNA^{Gly}$ are shown in red, green and orange boxes, respectively.

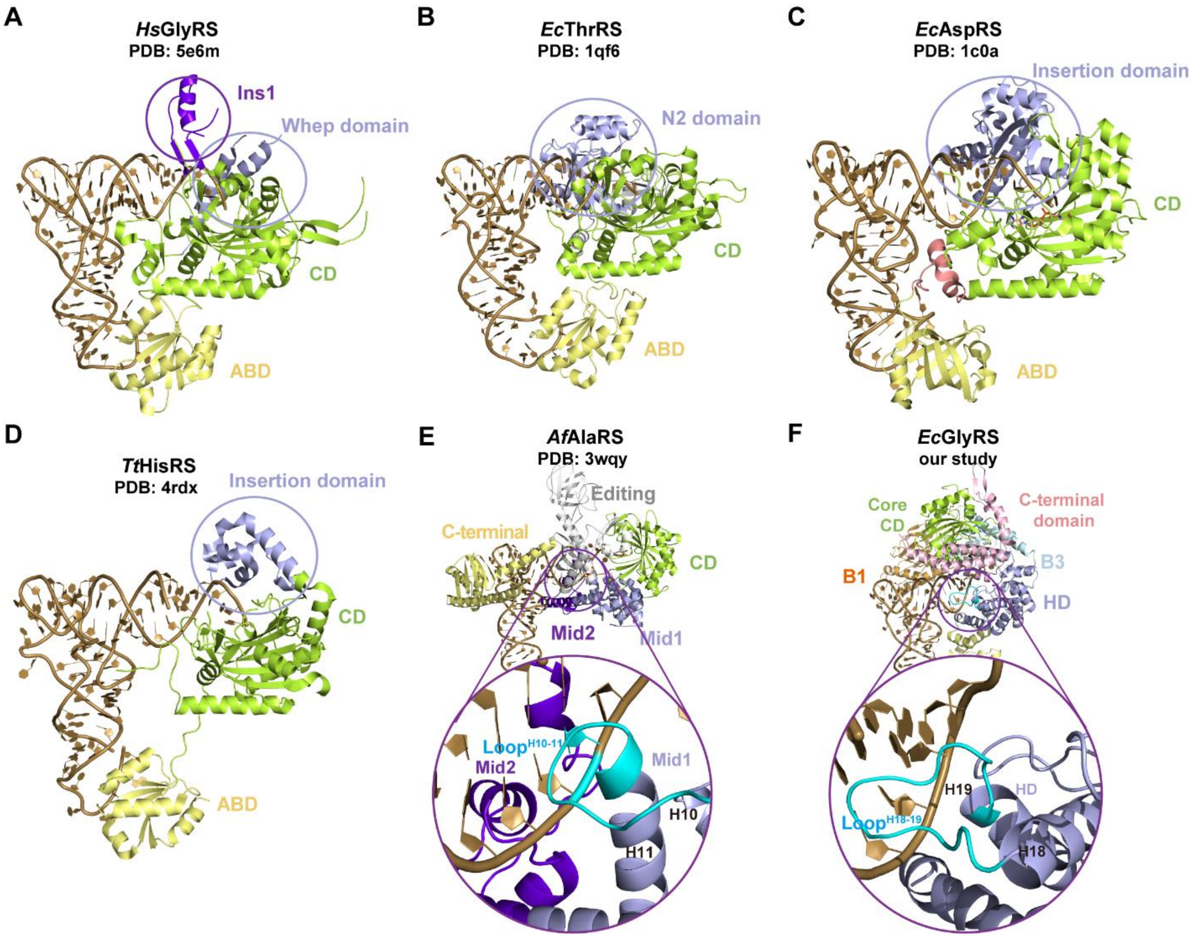

Fig. S7. Protein sequences beyond aminoacylation domain facilitate to recognize the acceptor stems and 3' termini of tRNAs in many class II synthetases. (A) In *Hs*GlyRS (PDB code 5e6m), the Ins1 and WHEP domains contact the minor groove of the acceptor stem and 3' terminus of $tRNA^{Gly}$, respectively. **(B)** In ThrRS (PDB code 1qf6), the N2 domain contacts the minor groove of the acceptor stem of $tRNA^{Thr}$. **(C)** In AspRS (PDB code 1c0a), the insertion domain contacts the 3' terminus of $tRNA^{Asp}$. **(D)** In HisRS (PDB code 4rdx), the insertion domain contacts the 3' terminus of $tRNA^{His}$. **(E)** In AlaRS (PDB code 3wqy), the Mid2 subdomain (purple) and the loop (cyan) before helix 11(H11) of Mid1 subdomain (light blue) clamp the minor and major grooves of the acceptor stem of $tRNA^{Ala}$. **(F)** In orphan *Ec*GlyRS, the loop (cyan) similar to that of AlaRS from HD domain (light blue) clamps the major groove of the acceptor stem of $tRNA^{Gly}$. In **(A-F)**, the catalytic domains and ABDs are colored in green and yellow, respectively, and accessory sequences assisting the recognition of tRNA are colored in purple or light blue and circled.

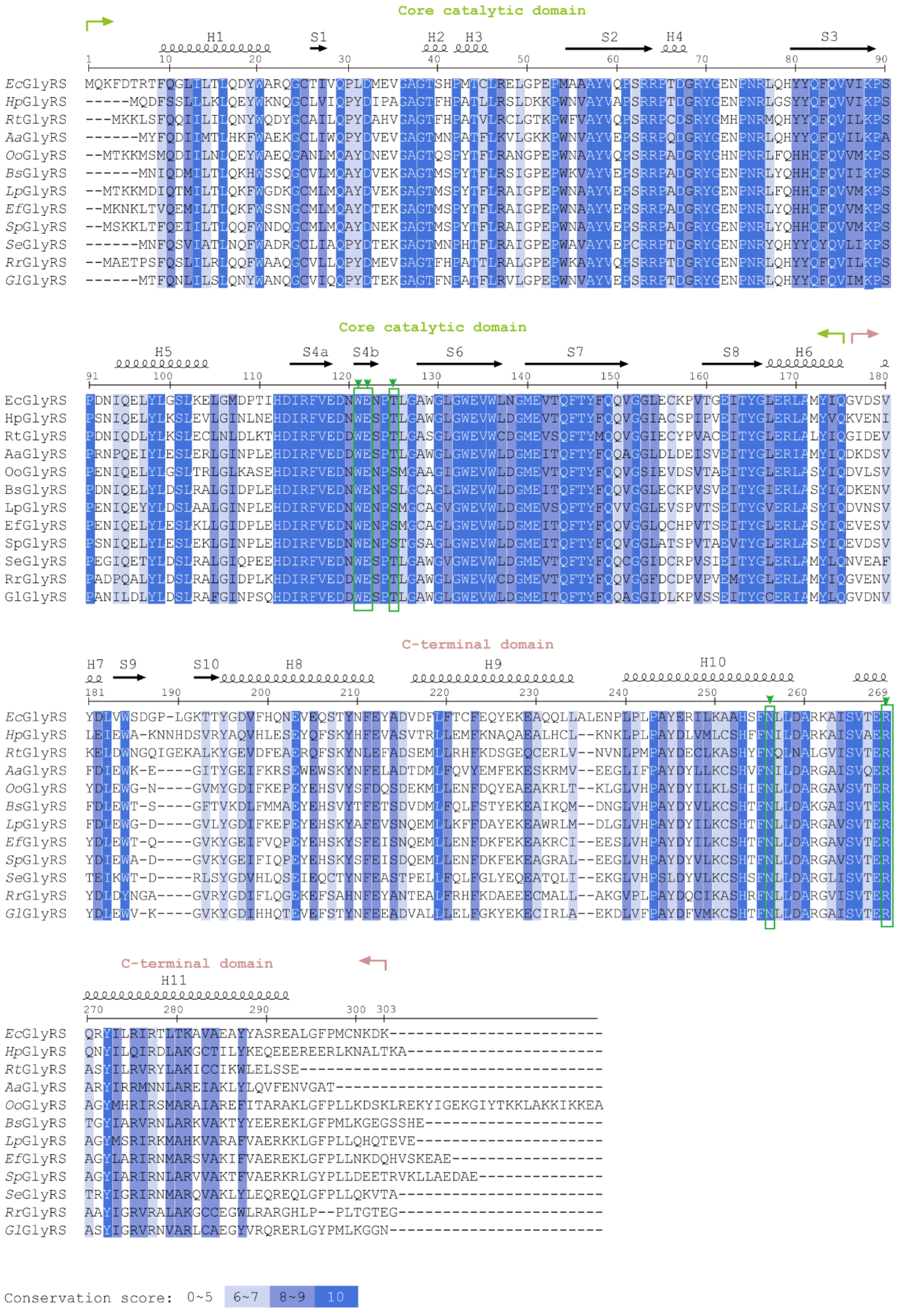

Core catalytic domain
H1 S1 H2 H3 S2 H4 S3
1 10 20 30 40 50 60 70 80 90
EcGlyRS MQKFDTRTFQGLILTLQDYWARQGCTIVQPLDMEVGAGTSHPMTCLRELGPEPMAAAYVQPSRRPTDGRYGENPNRLQHYYQFQVVIKPS
HpGlyRS -----MQDFSSLLLKLQEYWKNQGCLVIQPYDIPAGAGTFHPATLLRSLDKKPWNVAYVAPSRRPTDGRYGENPNRLGSYYQFQVVIKPS
RtGlyRS ---MKKLSFQQIILILQNYWQDYGCAILQPYDAHVGAGTFHPATVLRCLGTKPWFVAYVQPSRRPCDSRYGMHPNRMQHYYQFQVILKPS
AaGlyRS ------MYFQDIIMTLHKFWAEKGCLIWQPYDVEVGAGTMNPATFLKVLGKKPWNVAYVEPSRRPQDGRYGENPNRLQHYYQFQVILKPA
OoGlyRS --MTKKMSMQDIILNLQEYWAEQGANLMQAYDNEVGAGTQSPYTFLRANGPEPWNAAYVQPSRRPADGRYGHNPNRLFQHHQFQVVMKPS
BsGlyRS ------MNIQDMILTLQKHWSSQGCVLMQAYDVEKGAGTMSPYTFLRSIGPEPWKVAYVEPSRRPADGRYGENPNRLYQHHQFQVIIKPS
LpGlyRS --MTKKMDIQTMILTLQKFWGDKGCMLMQAYDVEKGAGTMSPYTFLRAIGPEPWNAAYVEPSRRPADGRYGENPNRLFQHHQFQVVMKPS
EfGlyRS --MKNKLTVQEMILTLQKFWSSNGCMLMQAYDTEKGAGTMSPYTFLRAIGPEPWNAAYVEPSRRPADGRYGENPNRLYQHHQFQVVMKPS
SpGlyRS --MSKKLTFQEIILTLQQFWNDQGCMLMQAYDNEKGAGTMSPYTFLRAIGPEPWNAAYVEPSRRPADGRYGENPNRLYQHHQFQVVMKPS
SeGlyRS ------MNFQSVIATINQFWADRGCLIAQPYDTEKGAGTMNPHTFLRAIGPEPWAVAYVEPCRRPTDGRYGENPNRYQHYYQYQVLIKPS
RrGlyRS --MAETPSFQSLILRLQQFWAAQGCVLLQPYDMEVGAGTFHPATTLRALGPEPWKAAYVQPSRRPTDGRYGENPNRLQHYYQFQVILKPS
GlGlyRS ------MTFQNLILSLQNYWANQGCVIQQPYDTEKGAGTFNPATFLRVLGPEPWNVAYVEPSRRPTDGRYGENPNRLQHYYQFQVIMKPS
Core catalytic domain
H5 S4a S4b S6 S7 S8 H6
91 100 110 120 130 140 150 160 170 180
EcGlyRS PDNIQELYLGSLKELGMDPTIHDIRFVEDNWENPTLGAWGLGWEVWLNGMEVTQFTYFQQVGGLECKPVTGEITYGLERLAMYIQGVDSV
HpGlyRS PSNIQELYLKSLEVLGINLNEHDIRFVEDNWESPTLGAWGLGWEVWLDGMEVTQFTYFQQVGGIACSPIPVEITYGLERLAMYVQKVENI
RtGlyRS PDNIQDLYLKSIECLNLDLKTHDIRFVEDDWESPTLGASGLGWEVWCDGMEVSQFTYMQQVGGIECYPVACEITYGLERLALYIQGIDEV
AaGlyRS PRNPQEIYIESLERLGINPLEHDIRFVEDDWESPTLGAWGLGWEVWLDGMEITQFTYFQQAGGLDLDEISVEITYGLERIAMYIQDKDSV
OoGlyRS PENIQELYLGSITRLGLKASEHDIRFVEDNWENPSMGAAGIGWEVWLDGMEVTQFTYFQQVGSIEVDSVTAEITYGLERLASYIQDVLSV
BsGlyRS PDNIQELYLDSIRALGIDPLEHDIRFVEDNWENPSLGCAGLGWEVWLDGMEITQFTYFQQVGGLECKPVSVEITYGIERLASYIQDKENV
LpGlyRS PENIQEYYLDSIAALGINPLEHDIRFVEDNWENPSMGCAGVGWEVWLDGMEVSQFTYFQVVGGLEVSPVTSEITYGVERLASYIQDVNSV
EfGlyRS PENIQELYLESIKLLGIDPLEHDIRFVEDNWENPSMGCAGLGWEVWLDGMEITQFTYFQQVGGLQCHPVTSEITYGLERLASYIQEVESV
SpGlyRS PSNIQELYLESIEKLGINPLEHDIRFVEDNWENPSTGSAGLGWEVWLDGMEITQFTYFQQVGGLATSPVTAEVTYGLERLASYIQEVDSV
SeGlyRS PEGIQETYLDSIRALGIQPEEHDIRFVEDNWESPTLGAWGVGWEVWLDGMEVTQFTYFQQCGGIDCRPVSIEITYGLERLAMYLQNVEAF
RrGlyRS PADPQALYLDSIRALGIDPLKHDIRFVEDDWESPTLGAWGLGWEVWCDGMEVTQFTYFQQVGGFDCDPVPVEMTYGLERLAMYIQGVENV
GlGlyRS PANILDLYLDSIRAFGINPSQHDIRFVEDDWESPTLGAWGLGWEVWLDGMEITQFTYFQQAGGIDLKPVSSEITYGCERIAMYLQGVDNV
C-terminal domain
H7 S9 S10 H8 H9 H10
181 190 200 210 220 230 240 250 260 269
EcGlyRS YDLVWSDGP-LGKTTYGDVFHQNEVEQSTYNFEYADVDFLFTCFEQYEKEAQQLLALENPLPLPAYERILKAAHSFNLLDARKAISVTER
HpGlyRS LEIEWA-KNNHDSVRYAQVHLESEYQFSKYHFEVASVTRLLEMFKNAQAEALHCL--KNKLPLPAYDLVMLCSHFFNILDARKAISVAER
RtGlyRS KELDWNGQIGEKALKYGEVDFEAERQFSKYNLEFADSEMLRHFKDSGEQCERLV--NVNLPMPAYDECLKASHYFNQLNALGVISVTER
AaGlyRS FDIEWK-E----GITYGEIFKRSEWEWSKYNFELADTDMLFQVYEMFEKESKRMV--EEGLIFPAYDYLLKCSHVFNILDARGAISVQER
OoGlyRS YDLEWG-N----GVMYGDIFKEPEYEHSVYSFDQSDEKMLLENFDQYEAEAKRLT--KLGLVHPAYDYILKLSHIFNLLDARGAVSVTER
BsGlyRS FDLEWT-S----GFTVKDLFMMAEYEHSVYTFETSDVDMLFQLFSTYEKEAIKQM--DNGLVHPAYDYVLKCSHTFNLLDAKGAISVTER
LpGlyRS FDLEWG-D----GVLYGDIFKEPEYEHSKYAFEVSNQEMLKKFFDAYEKEAWRLM--DLGLVHPAYDYILKCSHTFNLLDARGAVSVTER
EfGlyRS YDLEWT-Q----GVKYGEIFVQPEYEHSKYSFEISNQEMLLENFDKFEKEAKRCI--EESLVHPAYDYILKCSHTFNLLDARGAVSVTER
SpGlyRS YDIEWA-D----GVKYGEIFIQPEYEHSKYSFEISDQEMLLENFDKFEKEAGRAL--EEGLVHPAYDYVLKCSHTFNLLDARGAVSVTER
SeGlyRS TEIKWT-D----RLSYGDVHLQSEIEQCTYNFEASTPELLFQLFGLYEQEATQLI--EKGLVHPSLDYVLKCSHSFNLLDARGLISVTER
RrGlyRS YDLDYNGA----GVRYGDIFLQGEKEFSAHNFEYANTEALFRHFKDAEEECMALL--AKGVPLPAYDQCIKASHRFNLLDARGVISVTER
GlGlyRS YDLEWV-K----GVKYGDIHHQTEVEFSTYNFEEADVALLLELFGKYEKECIRLA--EKDLVFPAYDFVMKCSHTFNLLDARGAISVTER
C-terminal domain
H11
270 280 290 300 303
EcGlyRS QRYILRIRTLTKAVAEAYYASREALGFPMCNKDK-----------------------
HpGlyRS QNYILQIRDLAKGCTILYKEQEEEREERLKNALTKA---------------------
RtGlyRS ASYILRVRYLAKICCIKWLELSSE---------------------------------
AaGlyRS ARYIRRMNNLAREIAKLYLQVFENVGAT-----------------------------
OoGlyRS AGYMHRIRSMARAIAREFITARAKLGFPLLKDSKLREKYIGEKGIYTKKLAKKIKKEA
BsGlyRS TGYIARVRNLARKVAKTYYEEREKLGFPMLKGEGSSHE-------------------
LpGlyRS AGYMSRIRKMAHKVARAFVAERKKLGFPLLQHQTEVE--------------------
EfGlyRS AGYLARIRNMARSVAKIFVAERERLGFPLLNKDQHVSKEAE----------------
SpGlyRS AGYIARIRNLARVVAKTFVAERKRLGYPLLDEETRVKLLAEDAE-------------
SeGlyRS TRYIGRIRNMARQVAKLYLEQREQLGFPLLQKVTA----------------------
RrGlyRS AAYIGRVRALAKGCCEGWLRARGHLP--PLTGTEG----------------------
GlGlyRS ASYIGRVRNVARLCAEGYVRQRERLGYPMLKGGN-----------------------
Conservation score: 0~5 6~7 8~9 10

Fig. S8. Sequence alignments of the α subunits from *Ec*GlyRS and homologs. Protein sequences of *Ec*GlyRS (UniProtKB ID: P00960), *Hp*GlyRS (UniProtKB ID: B5Z7W3), *Rt*GlyRS (UniProtKB ID: Q68VR3), *Aa*GlyRS (UniProtKB ID: O67081), *Oo*GlyRS (UniProtKB ID: Q04F71), *Bs*GlyRS (UniProtKB ID: P54380), *Lp*GlyRS (UniProtKB ID: Q038U2), *Ef*GlyRS (UniProtKB ID: Q831U2), *Sp*GlyRS (UniProtKB ID: B8ZL21), *Se*GlyRS (UniProtKB ID: Q31KD2), *Rr*GlyRS (UniProtKB ID: Q2RQ44), *Gl*GlyRS (UniProtKB ID: B3E622) were aligned using Clustal Omega program (*72*). The secondary structures corresponding to *Ec*GlyRS are shown above the sequences. The conservation scores were calculated by the program Jalview (*73*) and presented in various shades of blue. Residues participating in the recognition of the 3' CCA-end of $tRNA^{Gly}$ are shown green boxes, respectively.

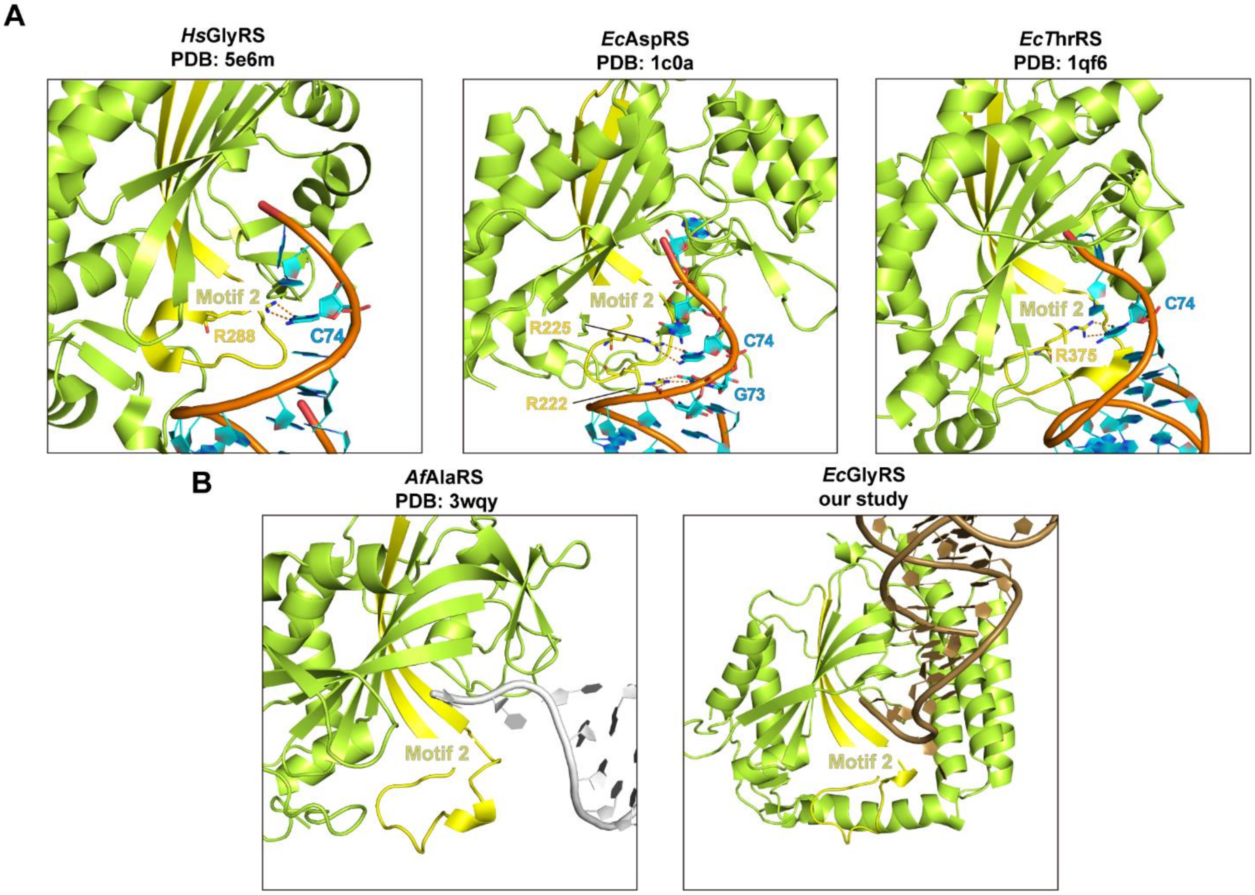

Fig. S9. The roles of class II signature motif 2 in the recognition of CCA-end of tRNAs for class II aaRSs. (A) The motif 2 contributes to stabilizing the 3' CCA-end of tRNA in typical class II aaRSs. The pyrimidine ring of C74 interacts with the conserved Arg in the motif 2 of *Hs*GlyRS (PDB code 5e6m), *Ec*AspRS (PDB code 1c0a) and *Ec*ThrRS (PDB code 1qf6). **(B)** In *Af*AlaRS (PDB code 3wqy) and orphan *Ec*GlyRS, their substrate tRNAs bind through an angle different to tRNAs of other class II aaRSs. As a result, the motif 2 of *Af*AlaRS and *Ec*GlyRS does not contact with 3' CCA-end of their tRNAs. In **(A-B)**, the motif 2 sequences are colored in yellow, and polar interactions between the conserved Arg on motif 2 and the 3' CCA-end of tRNA are labeled in orange dashed lines.

Table S1. Statistics of X-ray diffraction data collection and structure refinement.

	*Ec*GlyRS·GlySA·tRNA^{Gly}
**PDB accession code**	7YSE
**Data collection**	
Resolution(Å)	50.00-2.91(3.06-2.91)[a]
Wavelength (Å)	0.979
Space group	$P222_1$
Cell dimensions	
a, b, c (Å)	72.71, 162.123, 324.09
α, β, γ (°)	90.0, 90.0, 90.0
Unique reflections	85473 (12313)
Redundancy	6.6 (7.1)
R_{merge}[b]	0.138 (0.749)
$CC_{1/2}$	0.990 (0.774)
Average I/σ(I)	9.4 (2.6)
Completeness (%)	100 (100)
**Refinement**	
Resolution (Å)	50.00-2.91
Reflections for refinement/test	80999/4393
R_{work}[c]/R_{free}[d]	0.230/0.260
RMSD bond (Å)	0.004
RMSD angle (°)	1.231
No. atoms	
Protein	14340
RNA	2952
Water	14
Ion	6
Ligand	56
Average B factors (Å^2)	86.0
Ramachandran plot (%)	
Favored	93.74
Allowed	5.83
Outliers	0.43

[a]Values in parentheses are for the highest resolution shell.

[b]$R_{merge} = \Sigma_h \Sigma_l \mid I(h)_l - \langle I(h)\rangle \mid / \Sigma_h \Sigma_l I(h)_l$, where $I(h)_l$ is the lth observation of the reflection h and $\langle I(h)\rangle$ is the weighted average intensity for all observations l of reflection h.

[c]$R_{work}=\sum_h \mid \mid F_{obs}(h) \mid - \mid F_{cal}(h) \mid \mid /\sum_h \mid F_{obs}(h) \mid$, where $F_{obs}(h)$ and $F_{cal}(h)$ are the observed and calculated structure factors for reflection h respectively.

[d]R_{free} was calculated as R_{work} using 5.0% of the reflections which were selected randomly and omitted from refinement.

REFERENCES AND NOTES

1. L. Lei, Z. F. Burton, Evolution of life on Earth: tRNA, aminoacyl-tRNA synthetases and the genetic code. *Life (Basel)* **10**, 21 (2020).

2. L. Ribas de Pouplana, The evolution of aminoacyl-tRNA synthetases: From dawn to LUCA. *Enzymes* **48**, 11–37 (2020).

3. G. Eriani, M. Delarue, O. Poch, J. Gangloff, D. Moras, Partition of tRNA synthetases into two classes based on mutually exclusive sets of sequence motifs. *Nature* **347**, 203–206 (1990).

4. L. Ribas de Pouplana, P. Schimmel, Aminoacyl-tRNA synthetases: Potential markers of genetic code development. *Trends Biochem. Sci.* **26**, 591–596 (2001).

5. S. N. Rodin, S. Ohno, Two types of aminoacyl-tRNA synthetases could be originally encoded by complementary strands of the same nucleic acid. *Orig. Life Evol. Biosph.* **25**, 565–589 (1995).

6. Y. Pham, L. Li, A. Kim, O. Erdogan, V. Weinreb, G. L. Butterfoss, B. Kuhlman, C. W. Carter, Jr., A minimal TrpRS catalytic domain supports sense/antisense ancestry of class I and II aminoacyl-tRNA synthetases. *Mol. Cell* **25**, 851–862 (2007).

7. S. N. Chandrasekaran, G. G. Yardimci, O. Erdogan, J. Roach, C. W. Carter Jr., Statistical evaluation of the Rodin-Ohno hypothesis: Sense/antisense coding of ancestral class I and II aminoacyl-tRNA synthetases. *Mol. Biol. Evol.* **30**, 1588–1604 (2013).

8. L. Martinez-Rodriguez, O. Erdogan, M. Jimenez-Rodriguez, K. Gonzalez-Rivera, T. Williams, L. Li, V. Weinreb, M. Collier, S. N. Chandrasekaran, X. Ambroggio, B. Kuhlman, C. W. Carter, Functional class I and II amino acid-activating enzymes can be coded by opposite strands of the same gene. *J. Biol. Chem.* **291**, 23830–23831 (2016).

9. C. W. Carter Jr., Cognition, mechanism, and evolutionary relationships in aminoacyl-tRNA synthetases. *Annu. Rev. Biochem.* **62**, 715–748 (1993).

10. L. Ribas de Pouplana, P. Schimmel, Two classes of tRNA synthetases suggested by sterically compatible dockings on tRNA acceptor stem. *Cell* **104**, 191–193 (2001).

11. T. Terada, O. Nureki, R. Ishitani, A. Ambrogelly, M. Ibba, D. Soll, S. Yokoyama, Functional convergence of two lysyl-tRNA synthetases with unrelated topologies. *Nat. Struct. Biol.* **9**, 257–262 (2002).

12. Y. Ju, L. Han, B. Chen, Z. Luo, Q. Gu, J. Xu, X. L. Yang, P. Schimmel, H. Zhou, X-shaped structure of bacterial heterotetrameric tRNA synthetase suggests cryptic prokaryote functions and a rationale for synthetase classifications. *Nucleic Acids Res.* **49**, 10106–10119 (2021).

13. W. Freist, D. T. Logan, D. H. Gauss, Glycyl-tRNA synthetase. *Biol. Chem. Hoppe Seyler* **377**, 343–356 (1996).

14. W. Xie, L. A. Nangle, W. Zhang, P. Schimmel, X. L. Yang, Long-range structural effects of a Charcot-Marie-Tooth disease-causing mutation in human glycyl-tRNA synthetase. *Proc. Natl. Acad. Sci. U.S.A.* **104**, 9976–9981 (2007).

15. D. T. Logan, M. H. Mazauric, D. Kern, D. Moras, Crystal structure of glycyl-tRNA synthetase from *Thermus thermophilus*. *EMBO J.* **14**, 4156–4167 (1995).

16. D. L. Ostrem, P. Berg, Glycyl transfer ribonucleic acid synthetase from *Escherichia coli*: Purification, properties, and substrate binding. *Biochemistry* **13**, 1338–1348 (1974).

17. J.-U. Dimas-Torres, A. Rodríguez-Hernández, M. I. Valencia-Sánchez, E. Campos-Chávez, V. Godínez-López, D.-E. Rodríguez-Chamorro, M. Grøtli, C. Fleming, A. Hernández-González, M. Arciniega, A. Torres-Larios, Bacterial glycyl tRNA synthetase offers glimpses of ancestral protein topologies (2021); www.biorxiv.org/content/10.1101/2021.08.20.456953v6.

18. S. N. Rodin, A. S. Rodin, On the origin of the genetic code: Signatures of its primordial complementarity in tRNAs and aminoacyl-tRNA synthetases. *Heredity (Edinb)* **100**, 341–355 (2008).

19. C. W. Carter Jr., P. R. Wills, Hierarchical groove discrimination by class I and II aminoacyl-tRNA synthetases reveals a palimpsest of the operational RNA code in the tRNA acceptor-stem bases. *Nucleic Acids Res.* **46**, 9667–9683 (2018).

20. N. H. Kwon, P. L. Fox, S. Kim, Aminoacyl-tRNA synthetases as therapeutic targets. *Nat. Rev. Drug Discov.* **18**, 629–650 (2019).

21. F. L. Rock, W. Mao, A. Yaremchuk, M. Tukalo, T. Crepin, H. Zhou, Y.-K. Zhang, V. Hernandez, T. Akama, S. J. Baker, J. J. Plattner, L. Shapiro, S. A. Martinis, S. J. Benkovic, S. Cusack, M. R. K. Alley, An antifungal agent inhibits an aminoacyl-tRNA synthetase by trapping tRNA in the editing site. *Science* **316**, 1759–1761 (2007).

22. L. F. Silvian, J. Wang, T. A. Steitz, Insights into editing from an ile-tRNA synthetase structure with $tRNA^{ile}$ and mupirocin. *Science* **285**, 1074–1077 (1999).

23. K. Shiba, P. Schimmel, H. Motegi, T. Noda, Human glycyl-tRNA synthetase. Wide divergence of primary structure from bacterial counterpart and species-specific aminoacylation. *J. Biol. Chem.* **269**, 30049–30055 (1994).

24. F. Juhling, M. Morl, R. K. Hartmann, M. Sprinzl, P. F. Stadler, J. Putz, tRNAdb 2009: Compilation of tRNA sequences and tRNA genes. *Nucleic Acids Res.* **37**, D159–162 (2009).

25. Y. I. Wolf, L. Aravind, N. V. Grishin, E. V. Koonin, Evolution of aminoacyl-tRNA synthetases–Analysis of unique domain architectures and phylogenetic trees reveals a complex history of horizontal gene transfer events. *Genome Res.* **9**, 689–710 (1999).

26. J. Castropichel, M. T. Garcialopez, F. G. Delasheras, A facile synthesis of ascamycin and related analogues. *Tetrahedron* **43**, 383–389 (1987).

27. X. Deng, X. Qin, L. Chen, Q. Jia, Y. Zhang, Z. Zhang, D. Lei, G. Ren, Z. Zhou, Z. Wang, Q. Li, W. Xie, Large conformational changes of insertion 3 in human glycyl-tRNA synthetase (hGlyRS) during catalysis. *J. Biol. Chem.* **291**, 5740–5752 (2016).

28. R. Sankaranarayanan, A. C. Dock-Bregeon, P. Romby, J. Caillet, M. Springer, B. Rees, C. Ehresmann, B. Ehresmann, D. Moras, The structure of threonyl-tRNA synthetase-tRNA(Thr) complex enlightens its repressor activity and reveals an essential zinc ion in the active site. *Cell* **97**, 371–381 (1999).

29. V. Biou, A. Yaremchuk, M. Tukalo, S. Cusack, The 2.9 A crystal structure of T. thermophilus seryl-tRNA synthetase complexed with tRNA(Ser). *Science* **263**, 1404–1410 (1994).

30. N. Moor, O. Kotik-Kogan, D. Tworowski, M. Sukhanova, M. Safro, The crystal structure of the ternary complex of phenylalanyl-tRNA synthetase with tRNAPhe and a phenylalanyl-adenylate analogue reveals a conformational switch of the CCA end. *Biochemistry* **45**, 10572–10583 (2006).

31. M. Varadi, S. Anyango, M. Deshpande, S. Nair, C. Natassia, G. Yordanova, D. Yuan, O. Stroe, G. Wood, A. Laydon, A. Zidek, T. Green, K. Tunyasuvunakool, S. Petersen, J. Jumper, E. Clancy, R. Green, A. Vora, M. Lutfi, M. Figurnov, A. Cowie, N. Hobbs, P. Kohli, G. Kleywegt, E. Birney, D. Hassabis, S. Velankar, AlphaFold protein structure database: Massively expanding the structural coverage of protein-sequence space with high-accuracy models. *Nucleic Acids Res.* **50**, D439–D444 (2022).

32. A. Yaremchuk, I. Kriklivyi, M. Tukalo, S. Cusack, Class I tyrosyl-tRNA synthetase has a class II mode of cognate tRNA recognition. *EMBO J.* **21**, 3829–3840 (2002).

33. M. Naganuma, S. Sekine, Y. E. Chong, M. Guo, X. L. Yang, H. Gamper, Y. M. Hou, P. Schimmel, S. Yokoyama, The selective tRNA aminoacylation mechanism based on a single G*U pair. *Nature* **510**, 507–511 (2014).

34. E. Krissinel, K. Henrick, Inference of macromolecular assemblies from crystalline state. *J. Mol. Biol.* **372**, 774–797 (2007).

35. E. A. Wagar, M. J. Giese, B. Yasin, M. Pang, The glycyl-tRNA synthetase of *Chlamydia trachomatis*. *J. Bacteriol.* **177**, 5179–5185 (1995).

36. N. Nameki, K. Tamura, H. Asahara, T. Hasegawa, Recognition of tRNA(Gly) by three widely diverged glycyl-tRNA synthetases. *J. Mol. Biol.* **268**, 640–647 (1997).

37. B. Y. Lin, P. P. Chan, T. M. Lowe, tRNAviz: Explore and visualize tRNA sequence features. *Nucleic Acids Res.* **47**, W542-W547 (2019).

38. X. Qin, X. Deng, L. Chen, W. Xie, Crystal structure of the wild-type human GlyRS bound with tRNA(Gly) in a productive conformation. *J. Mol. Biol.* **428**, 3603–3614 (2016).

39. S. Eiler, A. Dock-Bregeon, L. Moulinier, J. C. Thierry, D. Moras, Synthesis of aspartyl-tRNA(Asp) in *Escherichia coli*–A snapshot of the second step. *EMBO J.* **18**, 6532–6541 (1999).

40. Q. Tian, C. Wang, Y. Liu, W. Xie, Structural basis for recognition of G-1-containing tRNA by histidyl-tRNA synthetase. *Nucleic Acids Res.* **43**, 2980–2990 (2015).

41. M. I. Valencia-Sanchez, A. Rodriguez-Hernandez, R. Ferreira, H. A. Santamaria-Suarez, M. Arciniega, A. C. Dock-Bregeon, D. Moras, B. Beinsteiner, H. Mertens, D. Svergun, L. G. Brieba, M. Grotli, A. Torres-Larios, Structural insights into the polyphyletic origins of glycyl tRNA synthetases. *J. Biol. Chem.* **291**, 14430–14446 (2016).

42. D. Hipps, K. Shiba, B. Henderson, P. Schimmel, Operational RNA code for amino acids: Species-specific aminoacylation of minihelices switched by a single nucleotide. *Proc. Natl. Acad. Sci. U.S.A.* **92**, 5550–5552 (1995).

43. P. Kumar, A. Bhatnagar, R. Sankaranarayanan, Chiral proofreading during protein biosynthesis and its evolutionary implications. *FEBS Lett.*, (2022), **596**, 1615, 1627.

44. M. Sprinzl, F. Cramer, Site of aminoacylation of tRNAs from *Escherichia coli* with respect to the 2′- or 3′-hydroxyl group of the terminal adenosine. *Proc. Natl. Acad. Sci. U.S.A.* **72**, 3049–3053 (1975).

45. C. W. Carter Jr, P. R. Wills, Class I and II aminoacyl-tRNA synthetase tRNA groove discrimination created the first synthetase-tRNA cognate pairs and was therefore essential to the origin of genetic coding. *IUBMB Life* **71**, 1088–1098 (2019).

46. L. E. Leiva, A. Pincheira, S. Elgamal, S. D. Kienast, V. Bravo, J. Leufken, D. Gutierrez, S. A. Leidel, M. Ibba, A. Katz, Modulation of *Escherichia coli* translation by the specific inactivation of tRNA(Gly) under oxidative stress. *Front. Genet.* **11**, 856 (2020).

47. M. H. Mazauric, H. Roy, D. Kern, tRNA glycylation system from *Thermus thermophilus*. tRNAGly identity and functional interrelation with the glycylation systems from other phylae. *Biochemistry* **38**, 13094–13105 (1999).

48. A. Yaremchuk, M. Tukalo, M. Grotli, S. Cusack, A succession of substrate induced conformational changes ensures the amino acid specificity of *Thermus thermophilus* prolyl-tRNA synthetase: Comparison with histidyl-tRNA synthetase. *J. Mol. Biol.* **309**, 989–1002 (2001).

49. B. Delagoutte, D. Moras, J. Cavarelli, tRNA aminoacylation by arginyl-tRNA synthetase: Induced conformations during substrates binding. *EMBO J.* **19**, 5599–5610 (2000).

50. S. Fukai, O. Nureki, S. Sekine, A. Shimada, J. Tao, D. G. Vassylyev, S. Yokoyama, Structural basis for double-sieve discrimination of L-valine from L-isoleucine and L-threonine by the complex of tRNA(Val) and valyl-tRNA synthetase. *Cell* **103**, 793–803 (2000).

51. K. Nakanishi, Y. Ogiso, T. Nakama, S. Fukai, O. Nureki, Structural basis for anticodon recognition by methionyl-tRNA synthetase. *Nat. Struct. Mol. Biol.* **12**, 931–932 (2005).

52. A. Palencia, T. Crepin, M. T. Vu, T. L. Lincecum, Jr., S. A. Martinis, S. Cusack, Structural dynamics of the aminoacylation and proofreading functional cycle of bacterial leucyl-tRNA synthetase. *Nat. Struct. Mol. Biol.* **19**, 677–684 (2012).

53. M. A. Rould, J. J. Perona, D. Söll, T. A. Steitz, Structure of *E. coli* glutaminyl-tRNA synthetase complexed with tRNA(Gln) and ATP at 2.8 A resolution. *Science* **246**, 1135–1142 (1989).

54. S. Hauenstein, C. M. Zhang, Y. M. Hou, J. J. Perona, Shape-selective RNA recognition by cysteinyl-tRNA synthetase. *Nat. Struct. Mol. Biol.* **11**, 1134–1141 (2004).

55. N. Shen, L. Guo, B. Yang, Y. Jin, J. Ding, Structure of human tryptophanyl-tRNA synthetase in complex with tRNATrp reveals the molecular basis of tRNA recognition and specificity. *Nucleic Acids Res.* **34**, 3246–3258 (2006).

56. B. Kuhle, M. Hirschi, L. K. Doerfel, G. C. Lander, P. Schimmel, Structural basis for shape-selective recognition and aminoacylation of a D-armless human mitochondrial tRNA. *Nat. Commun.* **13**, 5100 (2022).

57. H. H. Zhou, L. T. Sun, X. L. Yang, P. Schimmel, ATP-directed capture of bioactive herbal-based medicine on human tRNA synthetase. *Nature* **494**, 121–124 (2013).

58. J. Guo, B. Chen, Y. Yu, B. Cheng, Y. Cheng, Y. Ju, Q. Gu, J. Xu, H. Zhou, Discovery of novel tRNA-amino acid dual-site inhibitors against threonyl-tRNA synthetase by fragment-based target hopping. *Eur. J. Med. Chem.* **187**, 111941 (2020).

59. B. Y. Chen, S. T. Luo, S. X. Zhang, Y. C. Ju, Q. Gu, J. Xu, X. L. Yang, H. H. Zhou, Inhibitory mechanism of reveromycin A at the tRNA binding site of a class I synthetase. *Nat. Commun.* **12**, 1616 (2021).

60. Z. J. Cai, B. Y. Chen, Y. Yu, J. S. Guo, Z. T. Luo, B. Cheng, J. Xu, Q. Gu, H. H. Zhou, Design, synthesis, and proof-of-concept of triple-site inhibitors against aminoacyl-tRNA synthetases. *J. Med. Chem.* **65**, 5800–5820 (2022).

61. Y. Manickam, N. Malhotra, S. Mishra, P. Babbar, A. Dusane, B. Laleu, V. Bellini, M. A. Hakimi, A. Bougdour, A. Sharma, Double drugging of prolyl-tRNA synthetase provides a new paradigm for anti-infective drug development. *PLOS Pathog.* **18**, e1010363 (2022).

62. J. T. Zhou, Z. H. Huang, L. Zheng, Z. F. Hei, Z. Y. Wang, B. Yu, L. B. Jiang, J. Wang, P. F. Fang, Inhibition of plasmodium falciparum lysyl-tRNA synthetase via an anaplastic lymphoma kinase inhibitor. *Nucleic Acids Res.* **48**, 11566–11576 (2020).

63. Y. Nakamura, T. Gojobori, T. Ikemura, Codon usage tabulated from international DNA sequence databases: Status for the year 2000. *Nucleic Acids Res.* **28**, 292 (2000).

64. P. P. Chan, T. M. Lowe, GtRNAdb: A database of transfer RNA genes detected in genomic sequence. *Nucleic Acids Res.* **37**, D93–97 (2009).

65. C. Kao, M. Zheng, S. Rudisser, A simple and efficient method to reduce nontemplated nucleotide addition at the 3′ terminus of RNAs transcribed by T7 RNA polymerase. *RNA* **5**, 1268–1272 (1999).

66. F. Yu, Q. S. Wang, M. J. Li, H. Zhou, K. Liu, K. H. Zhang, Z. J. Wang, Q. Xu, C. Y. Xu, Q. Y. Pan, J. H. He, Aquarium: An automatic data-processing and experiment information management system for biological macromolecular crystallography beamlines. *J. Appl. Cryst.* **52**, 472–477 (2019).

67. C. Vonrhein, C. Flensburg, P. Keller, A. Sharff, O. Smart, W. Paciorek, T. Womack, G. Bricogne, Data processing and analysis with the autoPROC toolbox. *Acta Crystallogr. D Biol. Crystallogr.* **67**, 293–302 (2011).

68. A. Vagin, A. Teplyakov, Molecular replacement with MOLREP. *Acta Crystallogr. D* **66**, 22–25 (2010).

69. P. Emsley, B. Lohkamp, W. G. Scott, K. Cowtan, Features and development of coot. *Acta Crystallogr. D* **66**, 486–501 (2010).

70. G. N. Murshudov, A. A. Vagin, E. J. Dodson, Refinement of macromolecular structures by the maximum-likelihood method. *Acta Crystallogr. D Biol. Crystallogr.* **53**, 240–255 (1997).

71. V. B. Chen, W. B. Arendall, 3rd, J. J. Headd, D. A. Keedy, R. M. Immormino, G. J. Kapral, L. W. Murray, J. S. Richardson, D. C. Richardson, MolProbity: All-atom structure validation for macromolecular crystallography. *Acta Crystallogr. D Biol. Crystallogr.* **66**, 12–21 (2010).

72. F. Sievers, A. Wilm, D. Dineen, T. J. Gibson, K. Karplus, W. Li, R. Lopez, H. McWilliam, M. Remmert, J. Soding, J. D. Thompson, D. G. Higgins, Fast, scalable generation of high-quality protein multiple sequence alignments using Clustal Omega. *Mol. Syst. Biol.* **7**, 539 (2011).

73. A. M. Waterhouse, J. B. Procter, D. M. Martin, M. Clamp, G. J. Barton, Jalview Version 2–A multiple sequence alignment editor and analysis workbench. *Bioinformatics* **25**, 1189–1191 (2009).

74. R. Cain, R. Salimraj, A. S. Punekar, D. Bellini, C. W. G. Fishwick, L. Czaplewski, D. J. Scott, G. Harris, C. G. Dowson, A. J. Lloyd, D. I. Roper, Structure-guided enhancement of selectivity of chemical probe inhibitors targeting bacterial seryl-tRNA synthetase. *J. Med. Chem.* **62**, 9703–9717 (2019).
